# Multiple Levels of Organization in Amphiphilic Diblock Copolymers Based on Poly(γ-benzyl-l-glutamate) Produced by Aqueous ROPISA

**DOI:** 10.1021/acs.biomac.4c01657

**Published:** 2025-02-07

**Authors:** Marianna Spyridakou, Ioannis Tzourtzouklis, Robert Graf, Hannah Beauseroy, Colin Bonduelle, Sebastien Lecommandoux, George Floudas

**Affiliations:** † Department of Physics, University of Ioannina, P.O. Box 1186, 45110 Ioannina, Greece; ‡ Max Planck Institute for Polymer Research, Ackermannweg 10, 55128 Mainz, Germany; § University Bordeaux, CNRS, Bordeaux INP, LCPO, UMR 5629, F-33600 Pessac, France; ∥ University Research Center of Ioannina (URCI)-Institute of Materials Science and Computing, 45110 Ioannina, Greece

## Abstract

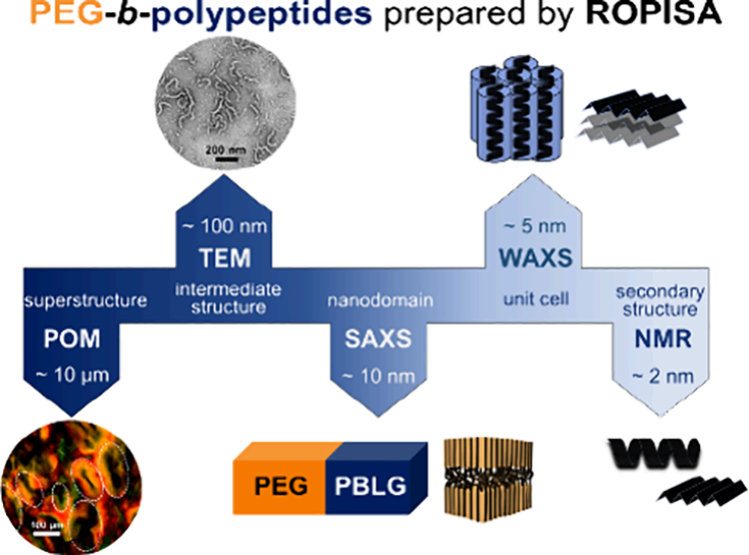

A recent method for producing amphiphilic block copolymers and nano-objects based on the ring-opening polymerization-induced self-assembly (ROPISA) in aqueous buffer is explored with respect to the tunability toward nanostructures. ROPISA gives rise to polypeptide copolymers with unprecedented levels of organization. By employing amphiphilic block copolymers of poly(ethylene glycol) (PEG) with the synthetic polypeptide poly(γ-benzyl-l-glutamate) (PBLG) and a combination of static (^13^C NMR, X-ray scattering, polarizing optical microscopy), thermodynamic (differential scanning calorimetry), and dynamic (dielectric spectroscopy) probes, we demonstrate a record of six levels of organization only found before in natural materials. These levels of organization could not be obtained in earlier morphology investigations of copolymers based on PEG and PBLG prepared by different methods. Furthermore, the type of NCA monomer (BLG-NCA vs Leu-NCA) and the solvent treatment method had an influence on the degree of segregation, the α-helical content, and the order-to-disorder transition temperature in the PEG-*b*-PBLG and PEG-*b*-PLeu copolymers.

## 1 Introduction

In the effort to design new functional materials with precisely controlled, at the synthesis level, internal dimensions and structures ranging from nanometer to macroscopic scales, synthetic polypeptides play an important role. The latter combine the complexity of biological macromolecules found in nature with the simpler synthetic polymers.^1−9^ In this respect, amphiphilic block copolymers based on the well-known synthetic polypeptide poly(γ-benzyl-l-glutamate) (PBLG)^10−14^ and the water-soluble poly(ethylene glycol) (PEG)^15^ have been synthesized and investigated with respect to the self-assembly.^6,16−19^ The presence of antagonistic tandem interactions between the two blocks (from one side, the tendency of PEG to crystallize by chain folding, and from the other side, the propensity of PBLG to form α-helical/β-sheet peptide secondary structures, on top of nanophase separation) gave rise to different levels of organization and structures key to the design of new functional materials.

A recent method for producing amphiphilic block copolymers and nano-objects is polymerization-induced self-assembly (PISA).^20,21^ The method affords the *in situ* one-step growth of a living amphiphilic polymer chain during its self-assembly into nanostructures. A variation of the method recently explored is based on the ring-opening polymerization-induced self-assembly (ROPISA) in aqueous buffer.^22−24^ It was employed with a PEG-NH_2_ macroinitiator and either benzyl-l-glutamate or l-leucine NCA monomers to synthesize well-defined amphiphilic block copolymers of PEG-*b*-PBLG and PEG-*b*-PLeu in a rapid way. The latter were found to stabilize anisotropic rod-like nanostructures. It was thought that the peptide secondary structures were responsible for the different nanostructures (α-helical/β-sheet for PBLG and PLeu, respectively).

Herein we employ the same diblock copolymers (PEG-*b*-PBLG and PEG-*b*-PLeu) and explore the self-assembly and polypeptide dynamics over different length and time scales, as a function of molar mass and for different annealing and solvent treatment protocols. By employing a combination of static (^13^C NMR, X-ray scattering, polarizing optical microscopy), thermodynamic (differential scanning calorimetry), and dynamic (dielectric spectroscopy) probes, we demonstrate a record of six levels of organization only found before in natural materials like tendon.^25^ They include (a) secondary structures of the polypeptides (α-helical/β-sheet), (b) the unit cell of PEG crystals, (c) the domain spacing of semicrystalline PEG, (d) the nanophase separation of unlike blocks, (e) an intermediate length-scale of rod-like objects (∼100 nm in size), and (f) distorted slowly growing anisotropic crystalline superstructures (∼100 μm). These structural motifs of the block copolymers prepared by the ROPISA method exceed both the structural integrity and the levels of organization found earlier in the same block copolymers^6,19^ but synthesized by different methods. We discuss herein the role of reduced interfacial mixing in polypeptide-based nanomaterials produced by ROPISA. Furthermore, we discuss the effect of solvent treatment (slow solvent evaporation versus freeze-drying versus organic solvent precipitation) on the degree of nanophase separation and peptide secondary structure.

## 2 Experimental Section

### 2.1 Synthesis

#### Typical Synthesis Procedure of Poly(ethylene glycol)-*b*-poly(γ-benzyl-l-glutamate) PEG_114_-*b*-PBLG_21_

In a glovebox, the NCA monomer of γ-benzyl-l-glutamate (150 mg, 0.57 mmol) was weighed in a Schlenk tube containing a magnetic stirring bar. The Schlenk is removed from the glovebox and cooled on ice. 4 mL of an ice-cooled solution of NaHCO_3_ 0.05 M containing the initiator PEG5k-NH2 (150 mg, 0.03 mmol, [M]/[I] = 19) is added to the BLG-NCA powder under a strong agitation (τs = 7%). The reaction is left to stir (1) first in an ice-cold water bath and (2) then at room temperature overnight. The opalescent dispersion obtained is then transferred to a 3.5 kDa dialysis membrane and dialyzed against deionized water for 2 days. An aliquot is kept for further microscopy imaging, and the remaining dispersion is lyophilized. A white powder is obtained with a yield of 85%. According to the method described by Grazon et al.,^23^ a PBLG degree of polymerization was calculated from ^1^H NMR (*N*_PBLG_ = 21; Figure S1, Supporting Information), and SEC then provided a number-average molecular weight *M*_n_ of 10900 g/mol (RI, PS calibration) and *Đ* = 1.06 (Figure S2, Supporting Information). TEM microscopy revealed a wormlike morphology (Figure S3).

^1^H NMR (400 MHz, CDCl_3_ + 15% TFA, δ, ppm): 7.85 (b, 1H, NH), 7.28 (b, 5H, Ar), 5.08 (q, 2H, CH_2_), 4.60 (b, 1H, CH), 3.70 (s, 4H, O–CH_2_CH_2_), 3.52 (s, 3H, CH_3_), 2.44 (b, 2H, CH_2_), 2.11–1.91 (b, 2H, CH_2_).

A part of the lyophilized powder was then used as such for further analysis (indicated below as the FD). Another part was solubilized in DMF, precipitated in diethyl ether, and dried under a vacuum overnight (indicated below as the OSP).

#### Typical Synthesis Procedure of Poly(ethylene glycol)-*b*-poly(γ-benzyl-l-glutamate) PEG_228_-*b*-PBLG_23_

This copolymer was prepared by the same method as described above for copolymer PEG_114_-*b*-PBLG_21_ except that the molar mass of PEG-NH2 was higher (PEG10k-NH2, 98 mg, 0.01 mmol, [M]/[I] = 19). The quantity of BLG-NCA was adjusted (49 mg, 0.19 mmol) as well as NaHCO_3_ 50 mM solution (2 mL) to keep the solid content at 7%. Yield: 83%.

^1^H NMR (400 MHz, CDCl_3_ + 15% TFA, δ, ppm): 7.85 (b, 1H, NH), 7.28 (b, 5H, Ar), 5.08 (q, 2H, CH_2_), 4.60 (b, 1H, CH), 3.70 (s, 4H, O–CH_2_CH_2_), 3.52 (s, 3H, CH_3_), 2.44 (b, 2H, CH_2_), 2.11–1.91 (b, 2H, CH_2_).

A PBLG degree of polymerization was calculated from ^1^H NMR (*N*_PBLG_ = 23; Figure S4), and SEC then provided a number-average molecular weight, *M*_n_, of 21400 g/mol (RI, PS calibration) and *Đ* = 1.05 (Figure S5). TEM microscopy revealed a mixture of spherical and worm-like morphology (Figure S6). Similarly, a part of the lyophilized powder was used as such for further analysis (FD). Another part was solubilized in DMF, precipitated in diethyl ether, and dried under a vacuum overnight (OSP).

#### Typical Synthesis Procedure of Poly(ethylene glycol)-*b*-poly(l-Leucine) PEG_114_-*b*-PLeu_28_

The same procedure was employed and adapted to the NCA monomer of l-leucine (Leu-NCA, 75 mg, 0.48 mmol). PEG114-NH2 (75 mg, 0.015 mmol, [M]/[I] = 32), dissolved in 2 mL of NaHCO_3_ 0.05 M, was used as the initiator (solid content τ = 7%). Yield: 78%.

According to the method described by Grazon et al.,^23^ a PLeu degree of polymerization was calculated from ^1^H NMR (*N*_PLeu_ = 28; Figure S7), and SEC then provided a number-average molecular weight *M*_n_ of 42000 g/mol (RI, calibration PMMA) and *Đ* = 1.05 (Figure S8). TEM microscopy revealed a mixture of spherical and worm-like morphology (Figure S9).

^1^H NMR (400 MHz, TFA-d, δ, ppm): 4.65 (b, 1H, CH), 3.85 (s, 4H, O–CH_2_CH_2_), 3.52 (s, 3H, CH_3_), 1.58 (b, 2H, CH_2_), 0.91–0.87 (b, 7H, −CH–(CH_3_)_2_).

Similarly, a part of the lyophilized powder was used for further analysis. Another part was solubilized in DMF, precipitated in diethyl ether, and dried under a vacuum overnight.

#### Typical Synthesis Procedure of Poly(γ-benzyl-l-glutamate) PBLG_25_

BLG NCA (1 g, 3.8 mmol, 50 equiv) was weighed in a previously flame-dried Schlenk in a glovebox under pure argon. The powder was then dissolved in 19 mL of anhydrous DMF (0.6 M) at room temperature. Hexylamine (10 μL, 0.08 mmol, 1 equiv) was added, and this was considered the starting time of the reaction. The solution was stirred for 3 days at room temperature under argon. FTIR allowed the monitoring of the reaction completion by monitoring the depletion of the bands at 1859 and 1787 cm^–1^, characteristic of the C=O stretching of NCAs. The polymer was then recovered by precipitation in diethyl ether twice and dried under high vacuum.

Yield: 670 mg; 67%, white powder. The degree of polymerization was calculated from ^1^H NMR (NPBLG = 25; Figure S10), and SEC provided a number-average molecular weight, *M*_n_, of 4000 g·mol^–1^ (RI, calibration PS) and *Đ* = 1.26 (Figure S11).

^1^H NMR (400 MHz, CDCl_3_ + 15% TFA, δ, ppm): 0.84 (t, 3H, CH_3_ hexylamine), 1.24 (br, 6H, CH_3_-(CH_2_)_3_- hexylamine), 1.45 (t, 2H, −CH_2_–CH_2_–NH- hexylamine), 1.89–2.09 (m, 2H, CH_2_), 2.42 (t, 2H, CH_2_, *J* = 6.8 Hz), 3.2 (m, 2H, −CH_2_–NH- hexylamine), 4.58 (m, 1H, CH), 5.06 (m, 2H, CH_2_O), 7.24–7.27 (m, 5H, ArH).

### 2.2 Methods of Polymer Characterization

^1^H NMR spectra were recorded at room temperature with a Bruker Avance 400 (400 MHz). CDCl_3_ and TFA-d were used as solvent, and signals were referred to the signal of residual protonated solvent signals.

#### Size Exclusion Chromatography (SEC)

Polymer molar masses were determined by SEC using dimethylformamide (DMF + LiBr 1 g/L) or hexafluoro-2-propanol (HFIP + 0.05% KTFA) as the eluent. Measurements in DMF were performed on an Ultimate 3000 system from ThermoFischer Scientific (Ilkirch, France) equipped with a diode array detector (DAD). The system also includes a multiangle light scattering detector (MALS) and differential refractive index detector dRI from Wyatt technology (Santa Barbara, CA, USA). Polymers were separated on three Shodex Asahipack gel columns [GF 310 (7.5 × 300 mm^2^), GF510 (7.5 × 300 mm^2^), exclusion limits from 500–300000 Da] at a flow rate of 0.5 mL/min. Columns temperature was held at 50 °C. Easivial kit of Polystyrene from Agilent (Santa Clara CA, USA) was used as calibration standard (*M*_n_ from 162 to 364000 Da). Measurements in HFIP were performed on an Ultimate 3000 system from Thermoscientific equipped with diode array detector DAD. The system also includes a multiangle light scattering detector MALS and differential refractive index detector dRI from Wyatt technology. Polymers were separated on two PL HFIP gel columns (300 × 7.5 mm; exclusion limits from 200 to 2000000 Da) at a flow rate of 0.8 mL/min. Column temperature was held at 40 °C. An easivial kit of PMMA from Agilent was used as the standard.

#### Transmission Electron Microscopy (TEM)

The images were recorded on a Hitachi H7650 microscope working at 80 kV. Samples were prepared by depositing a drop of 0.1 g/L NP dispersion onto a copper grid (200 mesh coated with carbon) and removing excess after 1 min. Grids were negatively stained with 1.2% uranyl acetate.

### 2.3 Small-Angle X-ray Scattering

Small-angle X-ray scattering (SAXS) measurements were made with the N8 Horizon vertical setup (Bruker), using a 50W CuKα radiation (IμS microfocus source with integrated MONTEL optics). The diffraction patterns were recorded on a VÅNTEC-500 2D detector (Bruker) at a sample–detector distance of 660 mm. The samples were placed in the form of a powder within borosilicate glass capillaries with a diameter of 1 mm. Intensity distributions as a function of the modulus of the scattering vector, *q* = (4π/λ)·sin(2θ/2), where 2θ is the scattering angle and λ = 0.154 nm is the wavelength, were obtained by radial averaging of the 2D data sets. Temperature-dependent measurements of 1 h long were made by slowly heating the samples from 303 to 423 K in 5 K steps, with 1 h equilibration time at each temperature, and subsequent cooling aimed at obtaining the disorder-to-order temperature.

### 2.4 Wide-Angle X-ray Scattering

Wide-angle X-ray scattering (WAXS) measurements were performed with a D8 Advance Bruker diffractometer, CuKα (40 kV, 40 mA) radiation, equipped with a secondary beam graphite monochromator (λ = 1.54184 nm). The system employed a Bragg–Brentano geometry in an θ–θ configuration. Patterns were obtained over the range of 2θ from 2 to 60 deg in steps of 0.01 deg, and the rate was 2 s per step for all samples. The recorded intensity distributions are presented as a function of the modulus of the scattering vector. Scattering curves were taken at a temperature of 303 K.

### 2.5 Solid-State NMR

^13^C CP MAS NMR spectra have been recorded with a Bruker Avance III NMR console operating at 500.20 MHz ^1^H Larmor frequency at a 11.7 T Oxford-Instruments wide-bore NMR magnet using a commercial double-resonance CP-MAS probe supporting zirconia MAS NMR rotors with 2.5 mm out diameter at a 25 kHz magic angle spinning frequency. The rf-power has been adjusted on both channels, ^1^H and ^13^C, to a 100 kHz rf-nutation frequency. A 90–100% ramped CP-contact pulse was used on the ^1^H channel, in order to account for possible rf instability and off-resonance conditions, and the duration of the CP contact time was 1 ms. High power swept-frequency TPPM decoupling^29^ with 100 kHz rf-nutation frequency has been applied on the ^1^H channel during acquisition. The sample temperatures under fast MAS spinning conditions have been corrected for frictional heating in the air bearings using the temperature dependent chemical shift of lead nitrate.^30^ The conformation dependent NMR signals of the polypeptides have been assigned according to Shoji et al.^31^ The quantitative analysis of the NMR spectra has been performed spectral fitting using the DMfit software.^32^

### 2.6 Polarizing Optical Microscopy

A Zeiss Axioskop40 polarizing optical microscope equipped with a video camera in a crossed polarizer configuration was used. Poly(tetrafluoroethylene) with a thickness of 25 μm was used as a spacer. The kinetics of superstructure formation was investigated by performing *T*-jumps from high temperatures (above PEG melting) to different final crystallization temperatures where the growth of the axialites was followed. Subsequently, the system was heated with 1 K·min^–1^ and the apparent melting temperature of the superstructure was recorded.

### 2.7 Differential Scanning Calorimetry (DSC)

A Q2000 (TA Instruments) was used for the thermal analysis with respect to the crystallization and melting of PEG and the presence of glass temperatures (*T*_g_). The instrument was calibrated (including baseline calibration) for best performance in the specific temperature range and heating/cooling rate using a sapphire standard. Samples were sealed in an aluminum pan and an empty pan was used as the reference. The temperature protocol involved measurements on heating and subsequent cooling with a rate of 10 K·min^–1^ and in a temperature range between 223 and 423 K. Temperature-modulated differential scanning calorimetry (TM-DSC) measurements were made the same Q2000 (TA Instruments) using cooling/heating rates in the range of 10^–1^ K·min^–1^ and oscillation periods from 20 to 150 s. The rate/period pairs used were as follows: 20 s, 10 K·min^–1^; 40 s, 5 K·min^–1^; 60 s, 3.3 K·min^–1^; 100 s, 2 K·min^–1^; 150 s, 1.3 K·min^–1^.

### 2.8 Dielectric Spectroscopy

Dielectric spectroscopy (DS) measurements as a function of temperature (*T*) were performed with a Novocontrol Alpha frequency analyzer. The temperature protocol involved “isobaric” measurements at atmospheric pressure within the temperature range from 183.15 to 423.15 K in steps of 5 K for frequencies in the range from 1 × 10^–2^ to 1 × 10^7^ Hz. All samples were prepared above the equilibrium melting temperature of PEG and under vacuum by pressing the electrodes to the spacer thickness where necessary. The sample cell consisted of two electrodes, 10 mm in diameter and 50 μm in thickness for all of the copolymers, the latter maintained by Teflon spacers. The complex dielectric function, ε* = ε′ – *i*ε′′, where ε′ is the real and ε′′ is the imaginary part, was obtained as a function of angular frequency, ω (=2π*f* = 1/τ) and temperature, i.e., ε*(Τ, ω).^33,34^ The analysis was made with the empirical equation of Havriliak and Negami (HN):
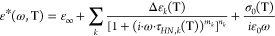
1where Δε_k_ is the dielectric strength, τ_HN,*k*_ is the H–N characteristic relaxation time, *m*_*k*_ and *n*_*k*_ (with limits 0.2 < *m*_*k*_, *mn*_*k*_ ≤ 1) describe, respectively, the symmetrical and asymmetrical broadening of the distribution of relaxation times, and the index *k* indicates the process under investigation. At lower frequencies, the dielectric loss sharply rises due to conductivity contribution as σ_0_/ε_0_ω, where σ_0_ is the dc-conductivity and ε_0_ (=8.854 × 10^–12^ F·m^–1^) is the permittivity of free space. From τ_ΗΝ,*k*_, the relaxation times at maximum loss, τ_max_, were obtained analytically from the HN equation as follows:
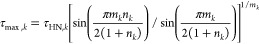
2

These relaxation times correspond to the relaxation times of the segmental processes. Except for the measured ε′′, the derivative of the real part of the dielectric permittivity, ε′  applicable for broad peaks was used in the analysis of the dynamic behavior.^35^

## 3 Results and Discussion

**Scheme 1:**
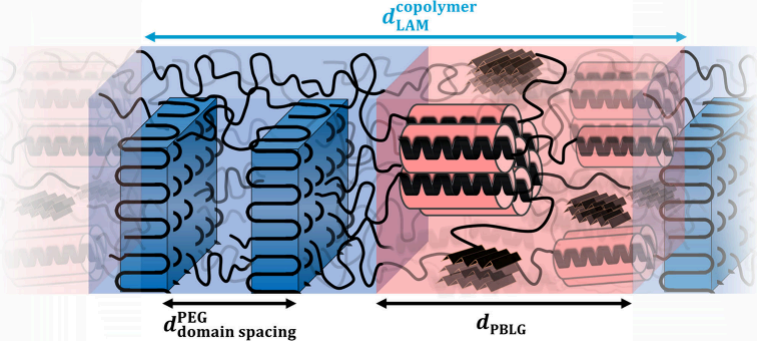
Schematic Representation of the Nanodomain Morphology of the PEG-*b*-PBLG Copolymer Prepared via the ROPISA Mechanism (By Following Either FD or OSP Processes) The blue color corresponds to the PEG block, while the red color indicates the PBLG block.

The aim of the study is 2-fold: first to compare the diblock copolymer morphology prepared by ROPISA with earlier studies^6,19^ on PEO-*b*-PBLG diblock copolymers and PBLG-*b*-PEO-*b*-PBLG triblock copolymers. To this end, we show that the level of organization produced by ROPISA is superior to that of earlier morphology investigations of copolymers based on PEG and PBLG prepared by other methods. Second, within the ROPISA method we explore the effects of solvent treatment and the effect of molar mass (Table 1). The question here is how solvent treatment (slow water evaporation vs freeze-drying vs organic solvent annealing as well as temperature) affects the degree of nanophase separation and peptide secondary structure. In the first protocol, the aqueous ROPISA polymerization was followed by a freeze-drying process (FD), while in the second protocol, a precipitation in organic solvent (OSP) was carried out right after freeze-drying. In general, slow water removal versus freeze-drying produces the same level of nanophase segregation and the same peptide secondary structure. However, OSP produced better equilibrated copolymers taking into account that this solvent may reduce the influence of the secondary structure coming from the aqueous process.^23^ The results are presented in five sections below addressing the: thermodynamic properties, nanophase segregation, secondary structure, superstructure formation, and molecular dynamics, each revealing the underlying complexity and multifaceted nature of the PEG-*b*-polypeptide copolymers prepared via the ROPISA mechanism. A schematic representation of the lamellar nanodomain morphology found in PEG-*b*-PBLG copolymers (by following either FD or OSP processes) is depicted in Scheme 1. Within each block, there are multiple levels of organization. In the PEG block, the monoclinic unit cell of PEG, the well-defined crystalline lamellar, and, at much higher length scales, the PEG overarching axialitic superstructures can be identified, while the polypeptide domain embeds the two secondary structures, α-helices and β-sheets, with the former further packed in a hexagonal lattice.

**Table 1:** Molecular Characteristics of the Investigated Copolymers

samples	*Đ*	*N*^PEG^	*N*^peptide^	*f*_PEG_^a^	*w*_PEG_
PEG_114_-*b*-PLeu_32_ (FD)^b^	1.05	114	32	0.60	0.63
PEG_114_-*b*-PLeu_32_ (OSP)^c^	1.05	114	32	0.60	0.63
PEG_114_-*b*-PBLG_19_ (FD)	1.06	114	19	0.52	0.49
PEG_114_-*b*-PBLG_19_ (OSP)	1.06	114	19	0.52	0.49
PEG_228_-*b*-PBLG_19_ (FD)	1.05	228	19	0.66	0.63
PEG_228_-*b*-PBLG_19_ (OSP)	1.05	228	19	0.66	0.63

a ρ_PEG_^26^ = 1.064 g·cm^–3^, ρ_PBLG_^27^ = 1.278 g·cm^–3^, ρ_PLeu_^28^ = 1 g·cm^–3^.

b FD = freeze-drying.

c OSP = organic solvent precipitation.

### 3.1 Thermodynamic Properties

**Figure 1:**
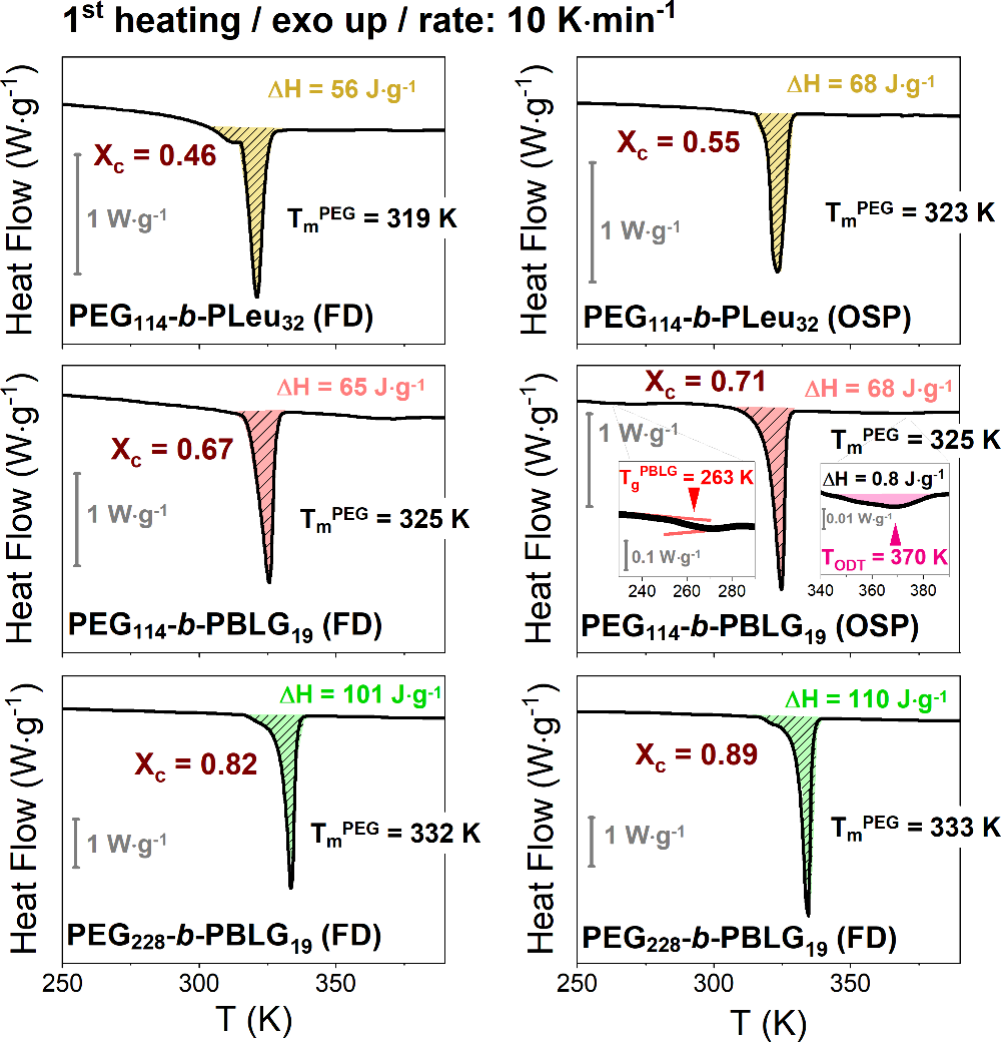
DSC traces of the copolymers, obtained during the first heating at a rate 10 K·min^–1^. The shadowed areas represent the heat of fusion for the semicrystalline PEG. Melting temperatures  and degrees of crystallinity (*X*_c_) are indicated. For PEG_114_-*b*-PBLG_19_ (OSP) the insets give the glass temperature of the PBLG block (left) and the order-to-disorder transition (with the heat of fusion) of the copolymer (right).

With DSC we address the degree of crystallinity, the melting temperatures, the possible liquid-to-glass temperatures, and the order-to-disorder transition temperature of the copolymers prepared by FD and OSP protocols. The DSC heating traces are presented in Figure 1, where an endothermic peak appears revealing the melting of PEG crystals. In all copolymers, the melting of the PEG block appears weakly dependent on the peptide block, as it is observed at slightly lower temperatures to that in the respective homopolymer (). However, the results differed for the crystallization temperature. Especially in PEG_114_-*b*-PBLG_19_ copolymer, the PEG compound crystallizes at 278 K (cooling traces shown in Figure S12), with a high degree of undercooling (Δ*T* ∼ 30 K), in contrast to bulk PEG (). The degree of crystallinity in the copolymers can be calculated from the heat of fusion, as , where Δ*Η*_∞_ = 196 J·g^–1^.^36^ The results are summarized in Table 2, along with the results of WAXS and NMR (to be discussed below).

**Table 2:** PEG Degree of Crystallinity, As Calculated from WAXS, ^13^C NMR, and DSC

samples	*X*_c_^WAXS^	*X*_c_^NMR^	*X*_c_^DSC^
PEG_114_-*b*-PLeu_32_ (FD)	0.45	0.27	0.46
PEG_114_-*b*-PLeu_32_ (OSP)	0.51	0.49	0.55
PEG_114_-*b*-PBLG_19_ (FD)	0.59	0.57	0.67
PEG_114_-*b*-PBLG_19_ (OSP)	0.67	0.66	0.71
PEG_228_-*b*-PBLG_19_ (FD)	0.68	0.69	0.82
PEG_228_-*b*-PBLG_19_ (OSP)	0.78	0.80	0.89

A closer look at the DSC trace of PEG_114_-*b*-PBLG_19_ (Figure 1 insets or Figure S13) can provide information about additional thermodynamic transitions. At temperatures corresponding below the melting of PEG, the glass temperature of the PBLG block is evident (at 263 K), while at temperatures above PEG melting, a first-order transition is evident corresponding to the order-to-disorder transition at *T*_ODT_ = 370 ± 1 K (value is in agreement with the SAXS results below). An estimation of the (χ*N*)_ODT_ parameter can be obtained from^37−39^ Δ*H* = *RT*_ODT_*f*(1 – *f*)(χ*N*)_ODT_/*M*_n_, where Δ*Η* (=0.8 ± 0.2 J·g^–1^) is the heat of fusion of the ODT transition, *R* is the gas constant, *f* (=0.52) is the volume fraction of PEG, *N* (=133) is the total degree of polymerization, and *M*_n_ (=9160 g·mol^–1^) is the total molar mass. This estimate provides (χ*N*)_ODT_ = 10 ± 1 at the transition, which is in agreement (within the experimental error) with the MFT predictions for diblock copolymers.^40^ Overall, the investigation of the thermodynamics revealed an influence of the solvent treatment on the PEG degree of crystallinity. Samples prepared by the freeze-drying method display a consistently lower degree of crystallinity. In addition, one of the copolymers prepared by OSP did show an ODT at higher temperatures.

### 3.2 Nanophase Separation

**Figure 2:**
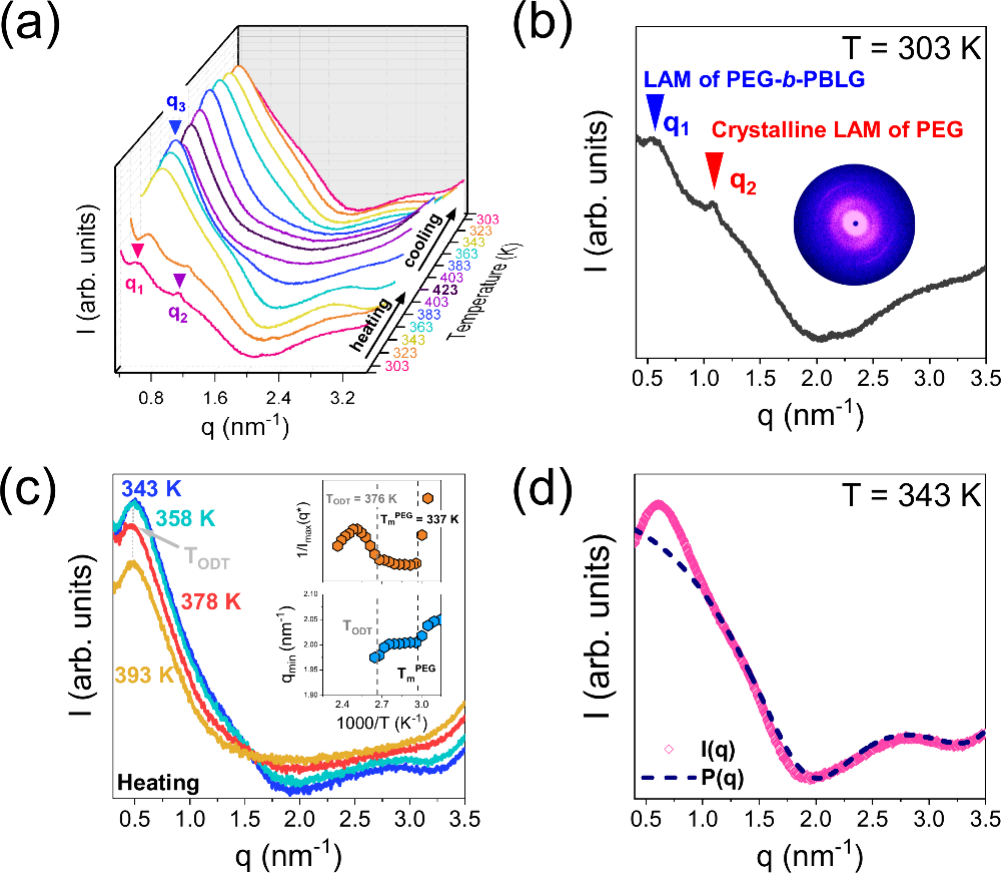
(a) SAXS patterns of PEG_114_-*b*-PBLG_19_ (OSP) block copolymer, during heating and subsequent cooling. The vertical arrows give the position of the Bragg reflections corresponding to the lamellar morphology of the copolymer (*q*_1_), the crystalline lamellar of PEG (*q*_2_), and the correlation hole scattering (*q*_3_). (b) SAXS pattern of PEG_114_-*b*-PBLG_19_ recorded at 303 K. The corresponding 2D pattern from the extracted fiber is also shown. (c) SAXS patterns at 343, 358, 378, and 393 K, indicating the characteristic order-to-disorder transition. The insets provide the inverse peak intensity and the *q*_min_ (position of the first minima in the form factor) as a function of inverse temperature. The dashed lines indicate the melting and order-to-disorder transition temperatures. (d) SAXS pattern of PEG_114_-*b*-PBLG_19_ recorded at 343 K. The blue dashed-line represents the Percus–Yevick approximation for cylindrically shaped objects (eq 7).

Precise information about the nanodomain morphology can be obtained by small-angle X-ray scattering. Some representative SAXS curves of PEG_114_-*b*-PBLG_19_ (OSP) are provided in Figure 2a (as all PEG-*b*-PBLG copolymers show similar SAXS results). Below the melting temperature of PEG, two Bragg reflections can be identified. The first intense peak, at *q*_1_ = 0.54 nm^–1^ (with periodicity *d*_1_ = 2π/*q* = 11.6 nm), reveals the formation of a lamellar (LAM) structure, indicating nanophase separated PEG/PBLG domains. The second sharper peak, at *q*_2_ = 1.1 nm^–1^ (*d*_2_ = 5.7 nm) reflects the domain spacing of semicrystalline PEG (Figure 2b). At temperatures above PEG melting, the copolymer exhibits a single broad peak at *q*_3_ ∼ 0.6 nm^–1^ (with a periodicity of ∼10.5 nm), indicative of correlation hole scattering.^41^ At *T* ∼ 378 ± 5 K, there is a discontinuous change of the peak intensity, in line with *T*_ODT_ obtained by DSC. The SAXS patterns exhibit some additional features at intermediate and higher *q*, with a distinct minimum at *q* ∼ 2 nm^–1^. They reflect the form factor of PBLG cylinders. In this case, the total scattered intensity is given by the product^42,43^
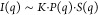
3where *P*(*q*) is the form factor and *S*(*q*) is the structure factor. The structure factor of the low-*q* interference peak associated with the intersphere correlations can be described by the Percus–Yevick approximation for cylindrically shaped objects, assuming that the interaction between two “particles” does not depend on particle size or orientation (monodisperse approximation) and is given by a hard-sphere potential as
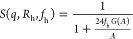
4where *A* = 2*qR*_h_, *R*_h_ and *f*_h_ are the effective interaction hard-sphere radius and volume fraction parameters describing the interference effects between the PBLG cylinders (i.e., the “particles”), and
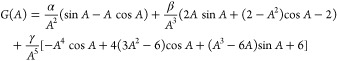
5Where,
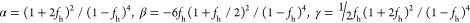
6

The form factor of monodisperse cylinders with a radius *R* and length *L* is defined as
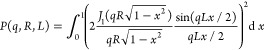
7where *J*_1_ is the first-order Bessel function. The simulation of the experimental scattering curve using the above theoretical model (Figure 2d) results in a set of four fitting parameters: *R* = 2.15 ± 0.02 nm, *L* = 3.0 ± 0.2 nm, *R*_h_ = 0.87 ± 0.05 nm, *f*_h_ = 0.48 (where *R*_h_ is the helix radius and *L* is the helix length). The value of the helix length, *L*, can be compared with the length of an ideal helix as ξ_helix_^ideal^ = 0.15 nm·19 repeat units = 2.85 nm (the length of an ideal helix is 0.15 nm per repeat unit).^13,14^ This suggests that the persistence length of the helices in the polypeptide block increases in the presence of PEG, compared to that of the homopolymer (). The experimental value of *L* being even longer than that of an ideal α-helix can associate with the reported α-to-PPII helix conversion for PBLG prepared in THF/water solutions.^44^ At temperatures *T* > 378 K, the broad peak continuously decreases in intensity, signaling the order-to-disorder (ODT) transition (Figure 2c), and the distinct minimum around *q* ∼ 2 nm^–1^ due to the form factor is lost (Figure 2c). This suggests that mixing of the different blocks at temperatures above the *T*_ODT_ eventually destabilizes the PBLG α-helices.

**Figure 3:**
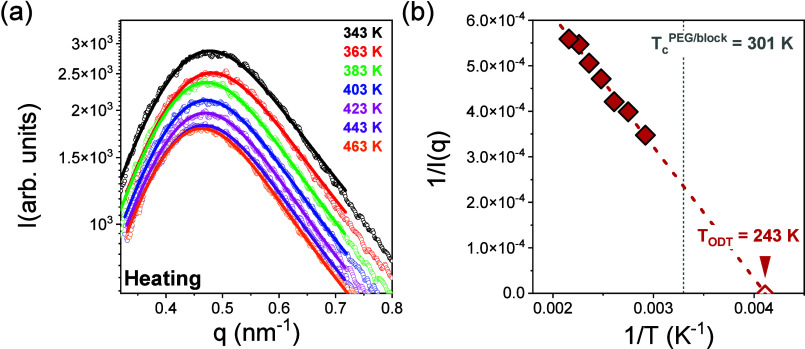
(a) SAXS patterns of PEG_114_-*b*-PLeu_32_ (OSP) at different temperatures, ranging from 343 to 463 K. A broad scattering maximum is evident, indicating scattering within the disordered state. The solid lines represent fits to the MFT. (b) Inverse peak intensity plotted vs inverse temperature. Extrapolating provides an estimate of the hypothetical order-to-disorder transition temperature (243 K). The vertical line gives the crystallization temperature of PEG.

The self-assembly in the second diblock system is very different. The SAXS data of PEG_114_-*b*-PLeu_32_ reveal mixing between the two blocks, even at lower temperatures. Within the disordered state, the structure factor can be described in the context of random phase approximation.^40^ The resulting fits are presented in Figure 3a, with the theory effectively describing the experimental data. The extracted interaction parameter displays a weak *T* dependence as χ = 0.07/*T* + 7·10^–6^. An estimation of the order-to-disorder temperature can be provided from the representation in Figure 3b. Notice that the *T*_ODT_ (=243 K) is significantly below the PEG block crystallization temperature (∼300 K), indicating that PEG crystallization initiates from the disordered melt state.

### 3.3 Secondary Structure

While scattering of X-rays at small angles (SAXS) can identify longer length scales, i.e., the nanodomain morphology of the copolymer and the crystalline lamellar of PEG, scattering at higher angles (WAXS) can provide the type and the organization of the peptide secondary structure, the PEG unit cell, and an independent measure of the PEG degree of crystallinity.

**Figure 4:**
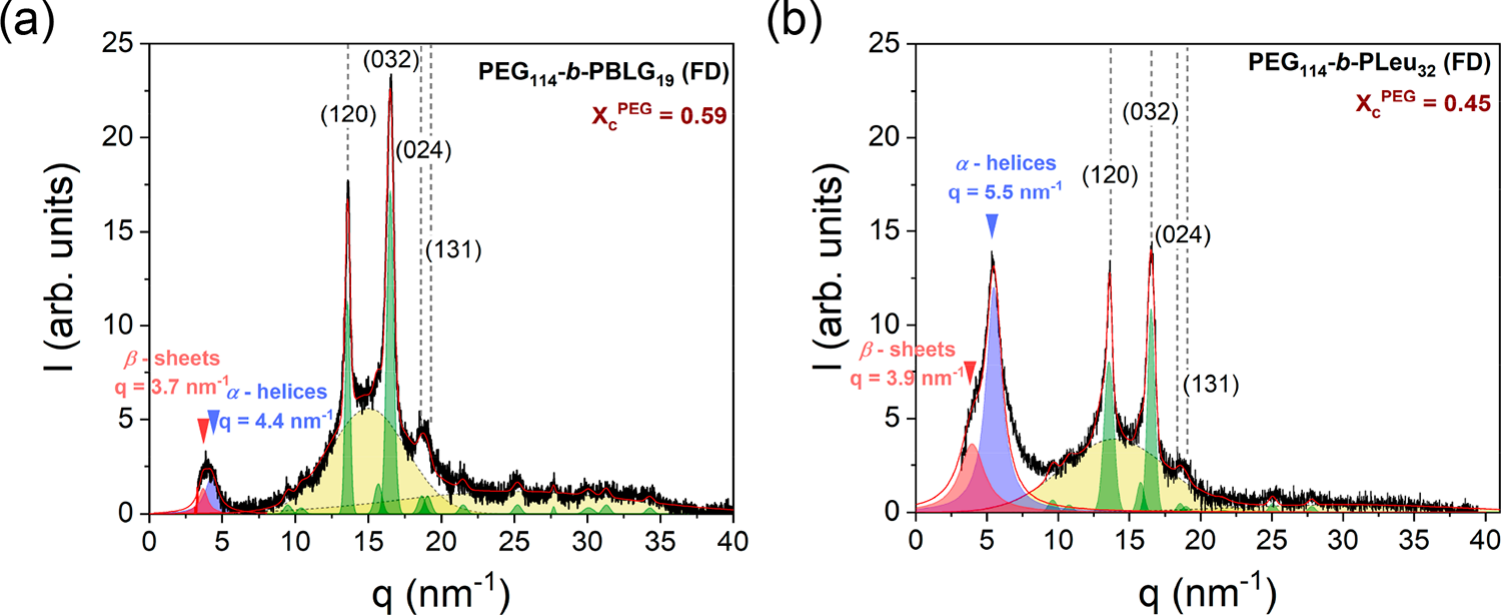
WAXS patterns of (a) PEG_114_-*b*-PBLG_19_ (FD) and (b) PEG_114_-*b*-PLeu_32_ (FD). At lower *q*, red arrows indicate the lamellar spacing of *b*-sheet secondary structure, while blue arrows give the position of the primary reflection from the weakly hexagonally packed cylinders composed from α-helical PBLG segments. At higher *q*, the short-dashed lines indicate the (*hkl*) indices of the Bragg reflections corresponding to the monoclinic unit cell of PEG, whereas the yellow areas give the contribution from the amorphous part.

Figure 4 gives the WAXS patterns of two representative copolymers, PEG_114_-*b*-PBLG_19_ (FD) and PEG_114_-*b*-PLeu_32_ (FD), bearing different polypeptide blocks. Figure S14 presents the remaining investigated copolymers. Starting from intermediate to higher *q*, both curves display several Bragg reflections, (120), (032), (024), (131) main reflections, corresponding to the ordinary monoclinic unit cell of PEG (unit cell parameters *a* = 0.81 nm, *b* = 1.30 nm, *c* = 1.95 nm, and β = 125.4°),^45^ while the amorphous halo reflects the semicrystalline nature of the copolymers. The degree of crystallinity of all investigated copolymers can be calculated as , where *f*_PEG_ is the volume fraction of PEG (Table 1), *I*_c_ is the intensity of all Bragg reflections associated with the monoclinic unit cell and *I*_a_ is the intensity of the amorphous halo (results from different methods are summarized in Table 2). The results show higher crystallinity in the OSP samples as compared to the FD samples. This effect can be attributed to the synthesis protocol of the copolymers. In the FD copolymer following the ROPISA mechanism, the chains grow and freeze in a less-optimum configuration as compared to the OSP copolymers. We will return to this point below with respect to the peptide secondary structure.

At lower *q* (*q* < 10 nm^–1^), both copolymers reveal features associated with the peptide secondary structure. Literature data from well-oriented PBLG^13,14^ reveal an α-helical secondary structure conformation with residues on a spiral pitch of 0.54 nm in a 18/5 helix (18 residues in 5 turns) with a repeat unit of *c* = 2.7 nm. The structure was ascribed to the paracrystalline form C; it consists of a periodic packing of α-helices in the direction lateral to the chain axis with a nematic-like paracrystalline order. The first strong equatorial reflection of PBLG at *q* = 4.4 nm^–1^ corresponds to the (10) reflection from a hexagonal unit cell of PBLG helices with a unit cell parameter of α = 1.65 nm. In the present copolymer PEG_114_-*b*-PBLG_19_ (Figure 4a), α-helices exist in the absence of long-range order, as revealed by the absence of higher order peaks. In addition, because of the low molar mass of PBLG, the presence of β-sheets at *q* ∼ 3.7 nm^–1^ is also evident.^13^ Respectively, for PEG_114_-*b*-PLeu_32_ (Figure 4b), the primary peak at *q* ∼ 5.5 nm^–1^ for the PLeu peptide block indicates the presence of weakly hexagonally packed α-helices (intercylinder distance of ∼1.32 nm), while β-sheets are shown from the weak Bragg peak at *q* ∼ 3.9 nm^–1^.^46^

**Figure 5:**
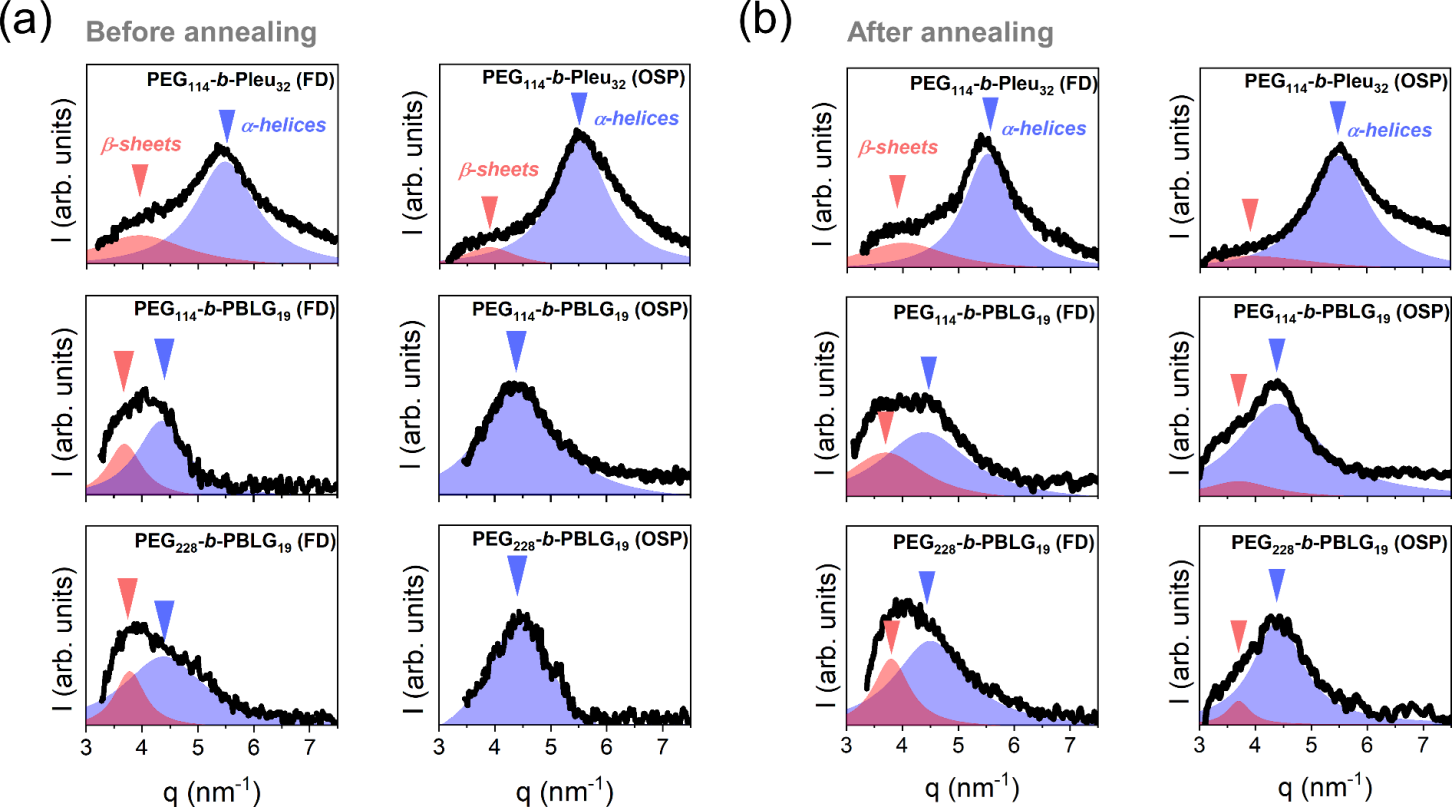
WAXS patterns of the copolymers (a) before and (b) after annealing for 1 day at 343 K. Red arrows identify the characteristic distance of β-sheets, while blue arrows indicate the position of the primary reflection associated with the α-helices.

As a next step, we investigate the effect of thermal annealing in Figure 5. Each secondary structure can be identified, as noted above, from its characteristic Bragg reflection, with the intensity of the peak corresponding to the relative β-sheet/α-helical fraction (in calculating fractions from WAXS assume only ordered secondary structures such as α-helices and β-sheets) in the peptide block (as shown in Table 3). Both before and after annealing, α-helices are the dominant secondary structures in all copolymers.

**Table 3:** Relative Fractions of α-Helices and β-Sheets in the Peptide Blocks of the Investigated Copolymers, as Calculated from WAXS (before and after Annealing) and Absolute Fractions of α-Helices, β-Sheets, and Random Coil, as Calculated from NMR (at 298 and 343 K)

	WAXS				NMR					
	β-sheets		α-helices		β-sheets		α-helices		random coil	
samples	b.a.^a^	a.a.^a^	b.a.^a^	a.a.^a^	298 K	343 K	298 K	343 K	298 K	343 K
PEG_114_-*b*-PLeu_32_ (FD)	0.33	0.34	0.67	0.66	0.35	0.38	0.45	0.41	0.20	0.21
PEG_114_-*b*-PLeu_32_ (OSP)	0.14	0.24	0.86	0.76	0.20	0.19	0.67	0.69	0.13	0.12
PEG_114_-*b*-PBLG_19_ (FD)	0.33	0.38	0.67	0.62	0.20	0.31	0.72	0.56	0.08	0.13
PEG_114_-*b*-PBLG_19_ (OSP)	0	0.13	1	0.87	0.02	0.13	0.93	0.73	0.05	0.14
PEG_228_-*b*-PBLG_19_ (FD)	0.22	0.31	0.78	0.69	0.19	0.32	0.81	0.57		0.11
PEG_228_-*b*-PBLG_19_ (OSP)	0	0.17	1	0.87	0.04	0.16	0.96	0.75		0.09

a b.a. = before annealing; a.a. = after annealing.

**Figure 6:**
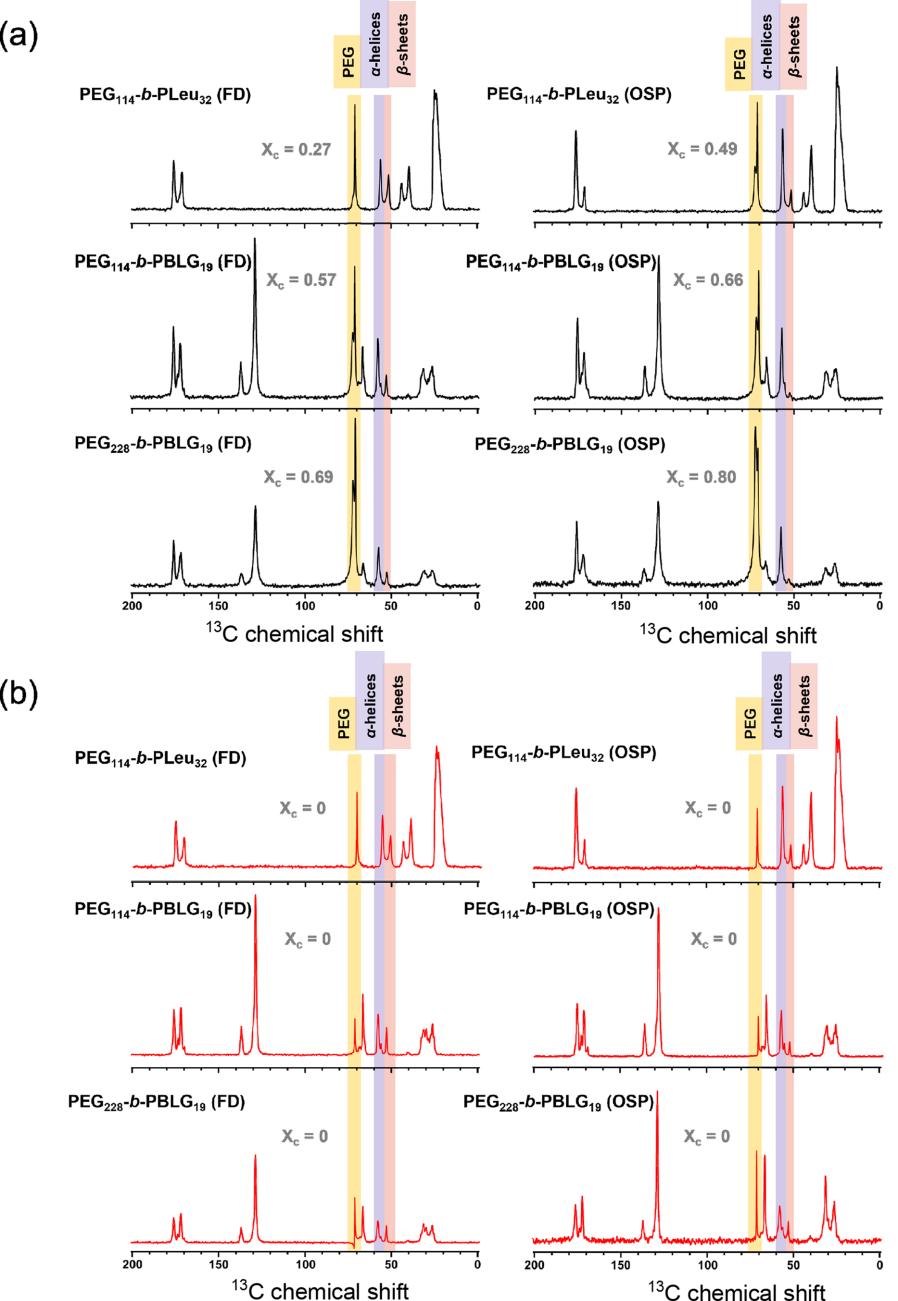
^13^C solid state NMR traces of the investigated copolymers at (a) 298 and (b) 343 K. The highlighted areas refer to the chemical shifts used for the calculations of the degree of crystallinity (PEG) and the fraction of peptide (PBLG, PLeu) secondary structure (see text).

One of the key probes for peptide secondary structure determination and PEG crystallinity determination is Solid State ^13^C NMR.^47−49^ The characteristic traces of the investigated copolymers are shown in Figure 6a at 298 K and in Figure 6b at 343 K. Starting from the PEG blocks, the intense resonances at δ ∼ 73 ppm and δ ∼ 71 ppm are assigned to the crystalline and amorphous signals, respectively.^46^ On the other hand, the resonances at δ ∼ 176 ppm (∼172 ppm) and δ ∼ 58 ppm (∼53 ppm), arising from the chemical shifts of the amide C=O and C_α_ carbon, respectively, reveal the formation of an α-helical (β-sheet) secondary structure in the peptide blocks. A distinct advantage of Solid State ^13^C NMR is the additional identification of the random coil conformations, which can be detected from the resonances at δ ∼ 53 ppm of the C_α_ carbon. A quantitative analysis of the intensity of the resonances can determine the degree of crystallinity (Table 2) and α-helical/β-sheet fractions (Table 3) in the copolymers. First, the α-helical content in the copolymers studied by slow water evaporation or freeze-drying is identical. Second, in all cases, the OSP samples exhibit higher relative α-helical fractions. For example, at 298 K, the α-helical fraction in the PEG_114_-*b*-PBLG_19_ copolymer is as high as 93%. This, at first sight, is surprising, as DMF can solubilize both blocks and possibly weaken the propensity for PBLG α-helices. Nevertheless, the results (Table 3) clearly show an increasing α-helical content probably due to the slow precipitation process. The lower α-helical content in the copolymers prepared by aqueous ROPISA can reflect the α-to-PPII helix transition reported for PBLG prepared in THF/water solutions.^44^ PPII is an extended helical conformation for PBLG as a result of the weaker intramolecular hydrogen bonds in THF/water solutions high in water content. It was further suggested that this conformational change at the level of secondary structure could ignite a macroscopic change of the self-assembled morphologies from fibers to particles.^44^

In addition to the peptide secondary structure, ^13^C NMR provides PEG crystallinity. Overall, the degree of crystallinity calculated from NMR shows excellent agreement with the WAXS results (Table 2). Potential discrepancies in the values, i.e. in the case of PEG_114_-*b*-PLeu_32_ (FD), arise from the restricted amorphous fractions in the PEG block and the size of its crystals. Concerning the secondary structure fractions, the comparison between the NMR results at 298 (343) K and the WAXS results before (after) annealing highlights the difference between the two probes. The ability to access the random coil fraction (*absolute fractions*) is now expressed as a “loss” in the α-helical content (Table 3). This underscores the complementary nature of NMR and WAXS results in providing a comprehensive understanding (from the different chemical shifts) of the amorphous and crystalline PEG and the peptide secondary structure. The secondary structure investigation by a combination of WAXS and solid state NMR revealed a higher α-helical content in the copolymers following the slow precipitation in organic solvent as opposed to the freeze-drying process, at the expense of the content of β-sheets and random coils. Furthermore, PEG-*b*-PLeu contains a high fraction of β-sheets when prepared via freeze-drying.

### 3.4 Superstructure Formation

**Figure 7:**
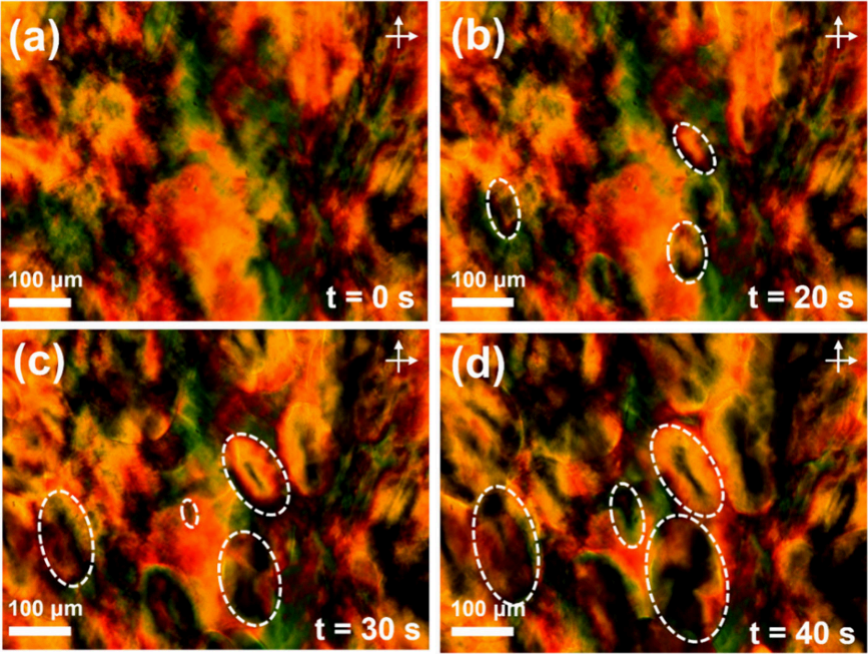
Representative POM images of the axialitic superstructures of PEG_114_-*b*-PBLG_19_ under isothermal conditions at *T* = 286 K, following jumps above the melting temperature, shown at different time intervals: (a) 0, (b) 20, (c) 30, and (d) 40 s. Dashed lines indicate the growing anisotropic superstructures.

The organization at a much higher length scale (superstructure) was studied by POM. Some representative POM images of PEG_114_-*b*-PBLG_19_ are shown in Figure 7. Interestingly, the superstructures deviate from the usual spherulitic shape of PEG bulk. The copolymer forms an axially crystalline morphology, demonstrating a distorted Maltese cross pattern.

**Figure 8:**
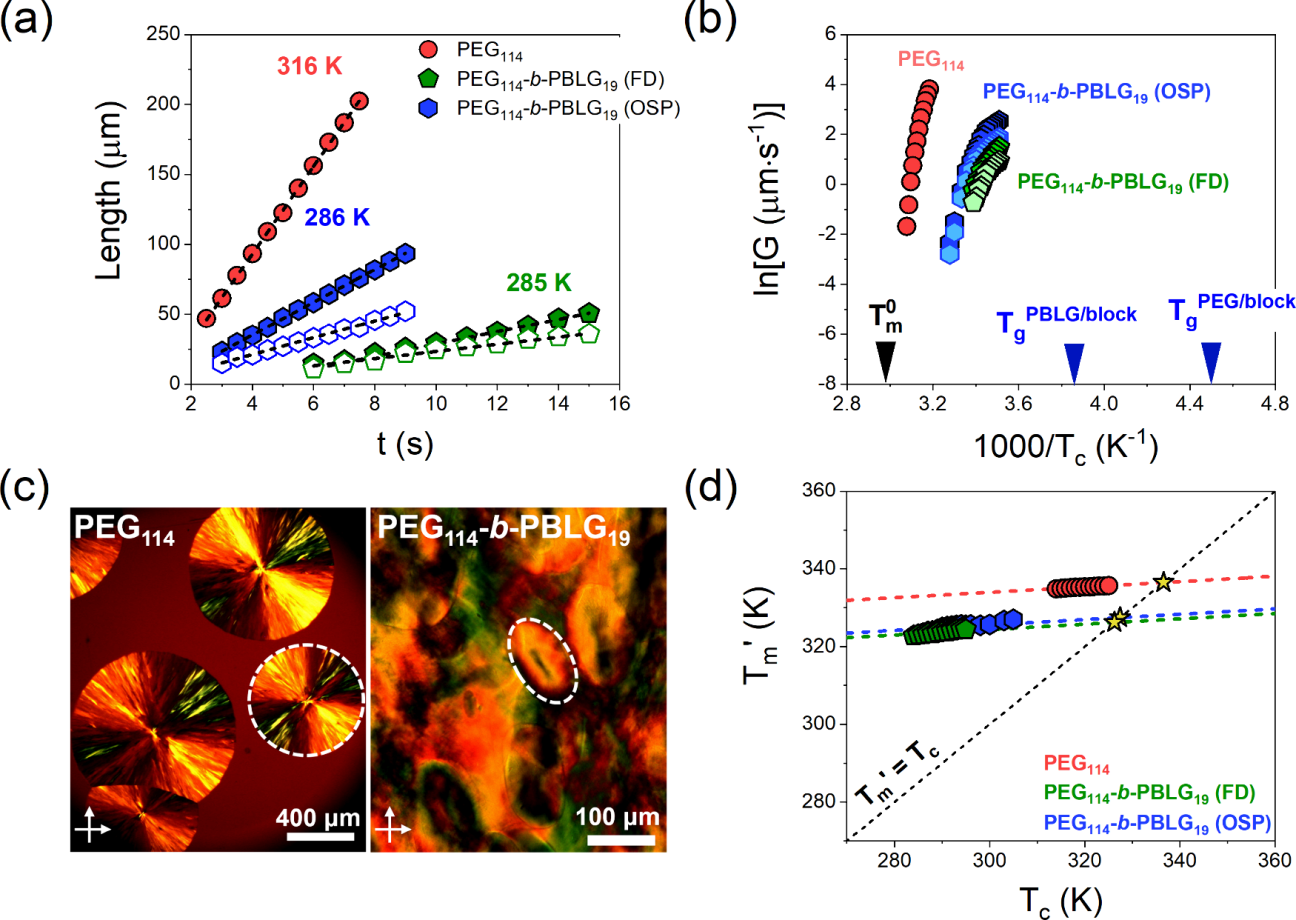
(a) Length of the long (filled symbols) and short (open symbols) axes (radius) of the PEG axialites (spherulites) as a function of time for PEG_114_-*b*-PBLG_19_ copolymers (bulk PEG_114_). (b) Growth rates of the superstructures as a function of the inverse crystallization temperature. Green and blue symbols correspond to FD and OSP PEG_114_-*b*-PBLG_19_ copolymers; dark and light colors represent the long and short axes, respectively. Red symbols represent the bulk PEG. Arrows indicate the equilibrium melting temperature and the glass temperatures for the PEG and PBLG block in the OSP prepared copolymer. (c) POM image, obtained under isothermal conditions for bulk PEG_114_ at 311 K and the PEG_114_-*b*-PBLG_19_ copolymer at 286 K. (d) Apparent melting temperatures plotted against the crystallization temperature (Hoffman–Weeks plot). Green and blue correspond to the PEG_114_-*b*-PBLG_19_ (FD), and PEG_114_-*b*-PBLG_19_ (OSP) copolymers, respectively, while red represents the bulk PEG. The slope (dashed lines) of the two copolymers were held constant (from the bulk PEG). Star symbols indicate the extrapolated equilibrium melting temperatures.

Subsequently, the kinetics of the superstructure formation were investigated (Figure 8) and different growth rates were obtained under isothermal conditions for different crystallization temperatures. Differences can be seen in both the temperature and the size of the axialites observed, in comparison to the spherulites found in the bulk PEG. The effect of thermodynamic confinement due to the presence of the PBLG block is 3-fold: First, the copolymers crystallize at lower temperatures (DSC results). Second, the inherent shape anisotropy of the PEG superstructures grows with time (Figure 8a). Third, the growth rates of the superstructures, when examined at a fixed temperature, are about 7 orders of magnitude slower than those of the PEG homopolymer (Figure 8b). Additionally, the Hoffman–Weeks plot shown in Figure 8d, reveals that the PEG crystals in the copolymers exhibit lower equilibrium melting temperatures, in comparison to PEG_114_, suggesting thermodynamic confinement and mixing. Concerning the PEG_114_-*b*-PLeu_32_ copolymer, formation of some anisotropic superstructures was also observed but the analysis of the growth rates was prohibited due to the extensive mixing between the two blocks (Figure 3).

### 3.5 Multiple Levels of Organization

**Figure 9:**
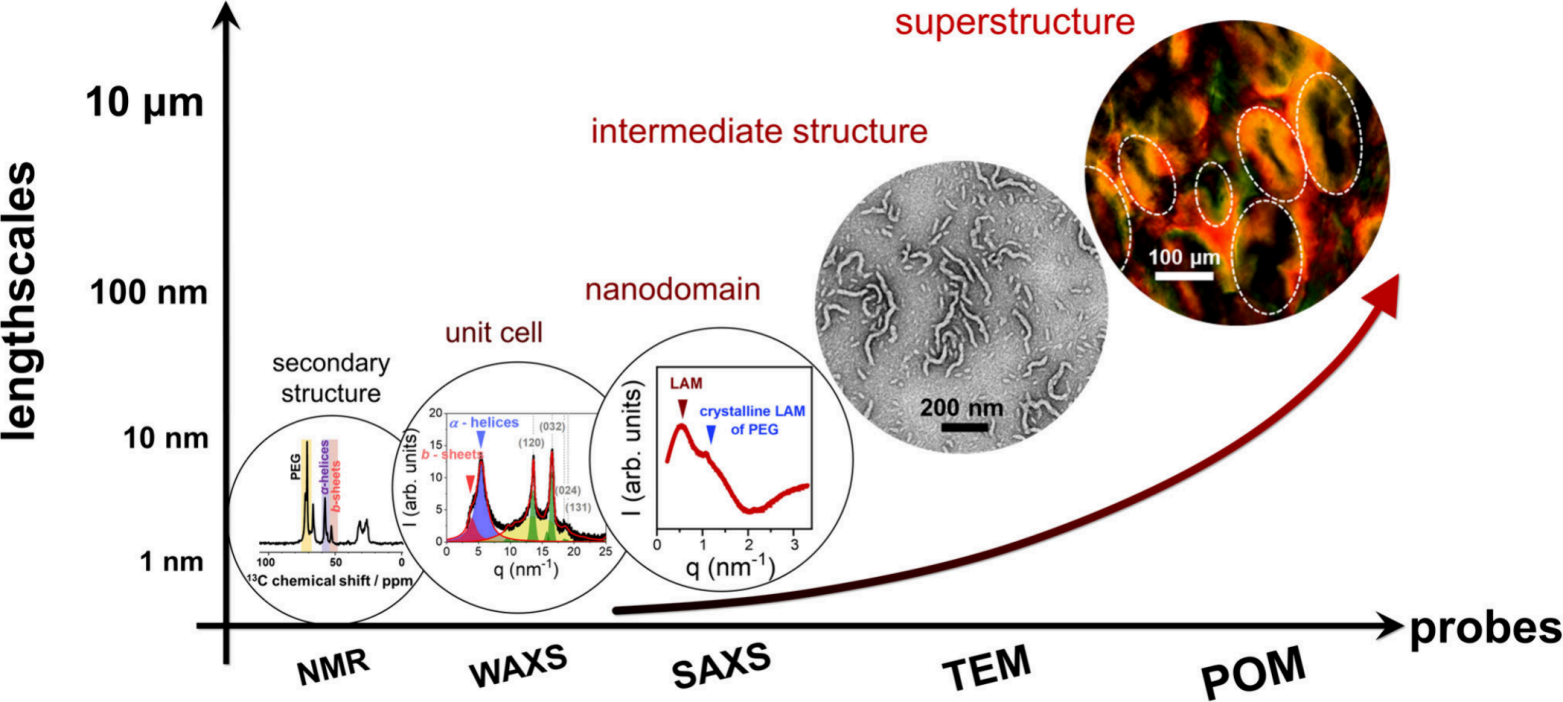
Schematic representation of the different length scales investigated for the PEG-*b*-PBLG copolymers prepared by ROPISA. Starting from smaller length scales they comprise; the secondary structures (α-helices and β-sheets), the PEG unit cell, the nanodomain morphology, an intermediate rod-like structure and the anisotropic superstructure can be identified, using a combination of probes (NMR, WAXS, SAXS, TEM/AFM, and POM).

The results from the structural investigation of the PEG-*b*-PBLG copolymers prepared by the ROPISA method can be summarized in a graphic plot, (Figure 9). It depicts the investigated length scales and the corresponding probes employed. First, NMR identified the peptide secondary structures (α-helices, β-sheets as well as some random coil configurations). Second, WAXS provided the unit cell of the semicrystalline PEG block. Third, SAXS measurements revealed three ordered “objects”: the form factor of PBLG α-helices, the domain spacing of the semicrystalline PEG, and the (lamellar) nanodomain morphology of the copolymer. Earlier TEM and AFM results in copolymers prepared by FP process provided some needle and wormlike intermediate structures.^22,23^ The nanoparticle morphology was linked to the secondary peptide secondary structure. For low molar mass PBLG a needle-like morphology appeared. Increasing the PBLG molar mass gave rise to wormlike morphologies as shown in Figure 9. At even longer length scales, POM documented the formation of some anisotropic superstructures. It was further shown that the growth of the latter superstructures was depended on the solvent treatment protocol. Overall, there exist six levels of organization in copolymers prepared by the ROPISA method imitating the multiple levels of organization found in natural materials.

Earlier studies on PEO-*b*-PBLG diblock copolymers and PBLG-*b*-PEO-*b*-PBLG triblock copolymers, the latter as a function of composition, revealed different morphologies comprising rods, lamellae, and broken lamellae with an increasing PBLG content. At a volume fraction of 0.34 < *f*_PBLG_ < 0.48 the prevailing morphology was lamellar in agreement with the present investigation.^19^ However, in both studies on PBLG-*b*-PEO-*b*-PBLG triblock copolymers^6,19^ the SAXS contrast was low, and a definite assignment of the different structures was not possible only by X-rays. Contrast this with the present SAXS investigation of the PEG-*b*-PBLG copolymers (Figure 2) where not only the lamellar nanodomain morphology but also the form factor of PBLG helices can be clearly obtained. This is an advantage of the ROPISA method that eliminates interfacial mixing giving rise to purer nanophases and nanostructures. Lastly, molecular dynamics provides a comprehensive understanding of the copolymers. While the static probes provided insights on the self-assembly of the copolymers over the several length scales, it is the molecular dynamic that can reveal the local and global peptide dynamics. Two processes were found: a segmental process with a strong temperature dependence followed by a slower process associated with the relaxation of helical parts. The dielectric strength of the slower process was employed in calculating the persistence length of the helices (Figure S15).

## 4 Conclusion

Aqueous ring-opening polymerization-induced self-assembly of NCA monomers with the hydrophilic macromolecular initiator α-amino-poly(ethylene glycol) controls unwanted water-induced NCA ring-opening (by the formation of protective micelles) and gives rise to amphiphilic block copolymers with several levels of organization. Six levels of organization were found in the PEG-*b*-PBLG copolymers prepared by ROPISA imitating the multiple levels of organization found in natural materials. They comprise: the lamellar nanodomain morphology of unlike blocks, the domain spacing of semicrystalline PEG within its monoclinic unit cell, the peptide secondary structures (α-helices and β-sheets) within the PBLG nanodomain, and at longer length scales some rod-like structures (typically ∼100 nm in size) and the strongly anisotropic superstructures of PEG crystals (typically ∼100 μm in size). These levels of organization could not be obtained in earlier morphology investigations of copolymers based on PEG and PBLG. Evidently, the ROPISA method eliminates interfacial mixing, giving rise to pure nanophases comprising PBLG domains with a high helical content. Subsequent, solvent annealing further gives rise to higher levels of organization within the nanodomains and an α-helical content as high as 93%.

Furthermore, the type of NCA monomer (BLG-NCA vs Leu-NCA) had an influence on the degree of segregation and the order-to-disorder transition temperature in the PEG-*b*-PBLG and PEG-*b*-PLeu copolymers. The latter have shown mixing of the unlike blocks at a temperature above the melting of PEG. In contrast, the low-*q* scattering of PEG-*b*-PBLG revealed a lamellar nanodomain morphology that could be described by the Percus–Yevick approximation for cylindrical shaped objects with a helix length approaching an “ideal” helix. At temperatures above the ODT, mixing of the unlike blocks resulted in destabilization of the PBLG α-helices.

Overall, the ROPISA method that combines a one-pot synthesis and self-assembly from aqueous solutions minimizes interfacial mixing and gives rise to polypeptide copolymers with unprecedented levels of organization. As a result, within the nanophases, the polypeptide block assumes secondary structures with high helical content.

## Supporting Information Available

Additional information on polymer synthesis, thermal properties by DSC, wide-angle X-ray scattering, and local and global dynamics by dielectric spectroscopy (PDF)
