# Supplementary material for: Multiple Levels of Organization in Amphiphilic Diblock Copolymers Based on Poly(γ-benzyl-l-glutamate) Produced by Aqueous ROPISA
Source: Biomacromolecules. 2025 Feb 7;26(3):1892–903. doi: 10.1021/acs.biomac.4c01657 (PMC11898064; doi:10.1021/acs.biomac.4c01657)
Supplement: Supplementary file 1 — bm4c01657_si_001.pdf [file bm4c01657_si_001.pdf]

SUPPORTING INFORMATION

Multiple Levels of Organization in Amphiphilic Diblock Copolymers Based on Poly(γ-benzyl-L-glutamate) Produced by Aqueous ROPISA

Marianna Spyridakou,[1] Ioannis Tzourtzouklis,[1] Robert Graf,[2]

Hannah Beauseroy,[3] Colin Bonduelle,[3*] Sebastien Lecommandoux,[3*]

George Floudas[1,2,4 *]

[1] *Department of Physics, University of Ioannina, P.O. Box 1186, 45110 Ioannina, Greece*

[2] *Max Planck Institute for Polymer Research, Ackermannweg 10, 55128 Mainz, Germany*

[3]*University Bordeaux, CNRS, Bordeaux INP, LCPO, UMR 5629, F-33600 Pessac, France.*

[4] *University Research Center of Ioannina (URCI)-Institute of Materials Science and Computing, 45110 Ioannina, Greece*

*: Corresponding author E-mails:

G.F. gfloudas@uoi.gr, ORCID: 0000-0003-4629-3817

C.B. colin.bonduelle@enscbp.fr, ORCID: 0000-0002-7213-7861

S.L. lecommandoux@enscbp.fr, ORCID: 0000-0003-0465-8603

A. Polymer synthesis

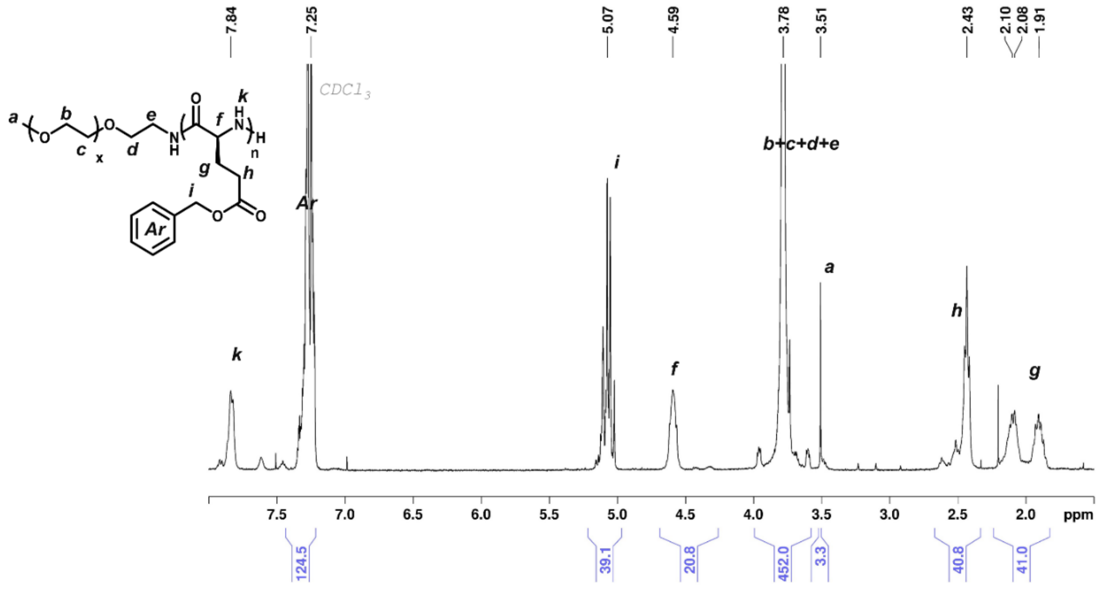

Figure S1. ^{1}H NMR spectrum of PEG_{114}-*b*-$PBLG_{19}$ in $CDCl_3$ + 15% TFA.

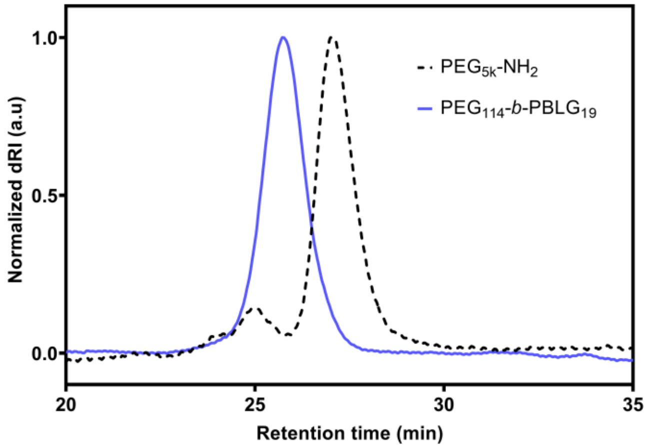

Figure S2. Steric exclusion chromatograms (SEC) of copolymer PEG_{114}-*b*-$PBLG_{19}$ compared to PEG_{5k}-NH_2 analyzed in DMF + 1 $g.L^{-1}$ LiBr (Differential refractive index detection, normalized).

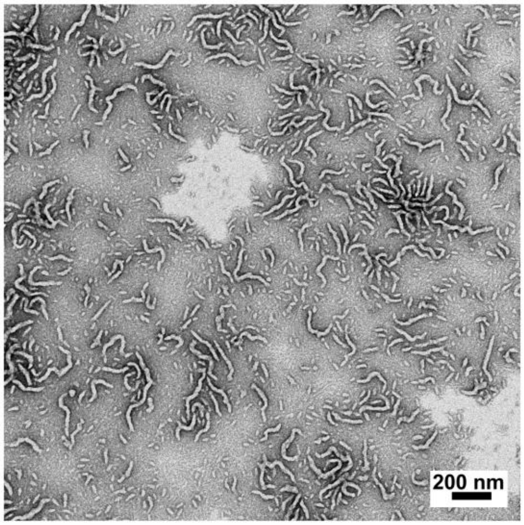

Figure S3. Representative transmission electronic microscopy (TEM) image of PEG$_{114}$-*b*-PBLG$_{19}$ nanoparticle suspensions, negatively stained with 1.2% uranyl acetate.

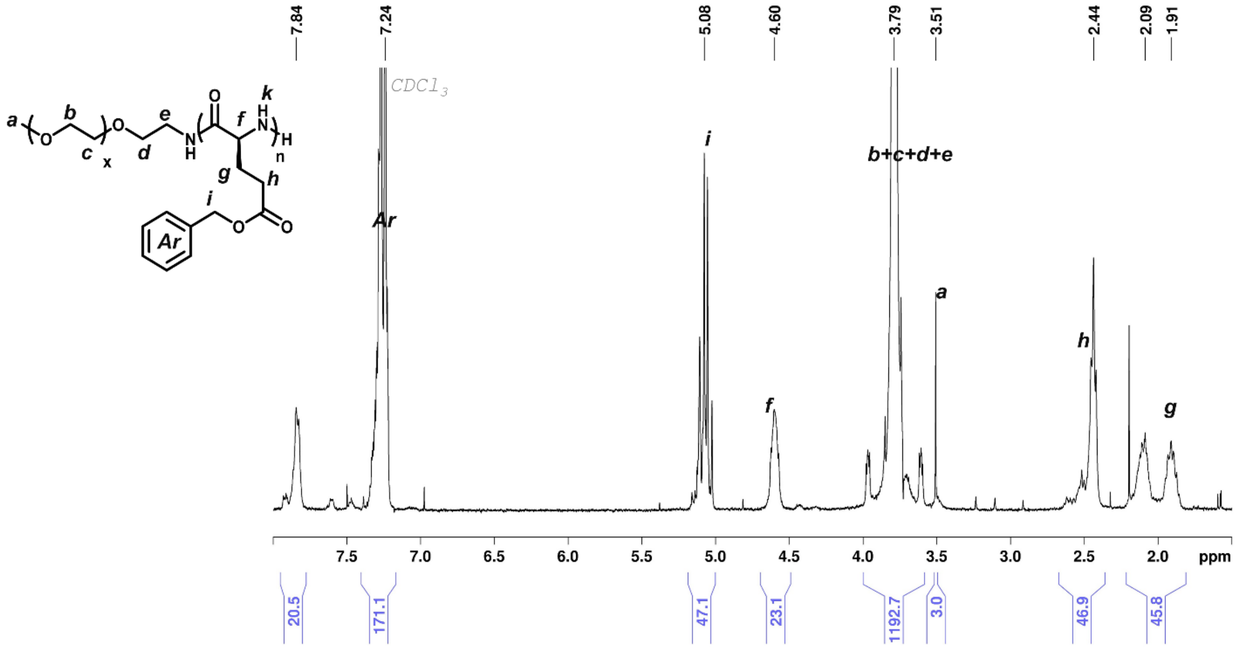

Figure S4. ^{1}H NMR spectrum of PEG$_{228}$-*b*-PBLG$_{19}$ in $CDCl_3$ + 15% TFA.

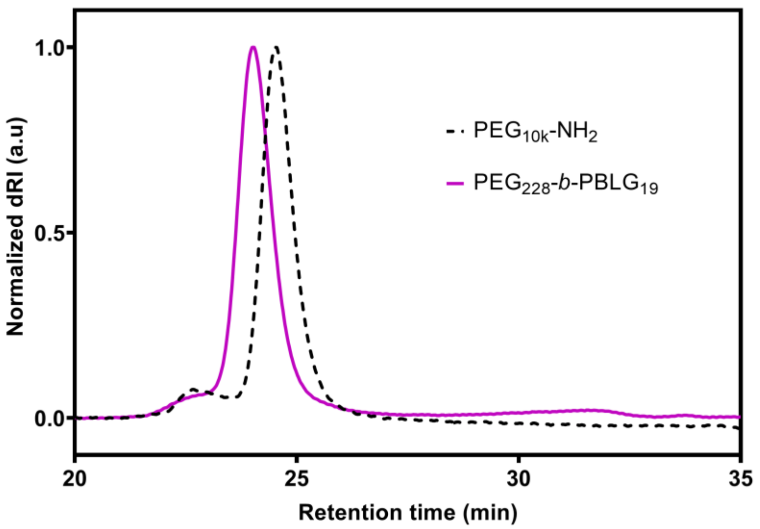

Figure S5. SEC traces of copolymer PEG_{228}-*b*-$PBLG_{19}$ compared to PEG_{10k}-NH_2 analyzed in DMF + 1 $g.L^{-1}$ LiBr (Differential refractive index detection, normalized).

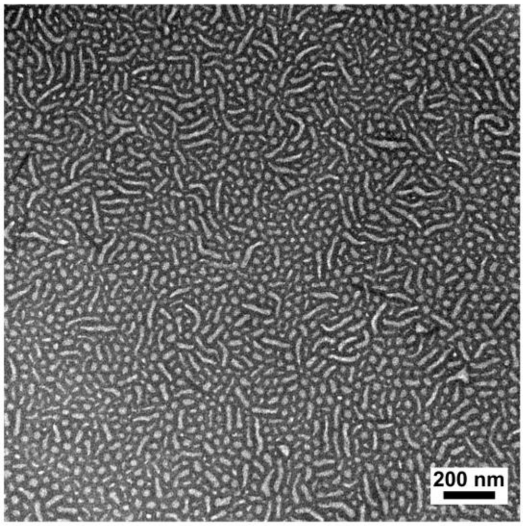

Figure S6. Representative TEM image of PEG_{228}-*b*-$PBLG_{19}$ nanoparticle suspensions, negatively stained with 1.2% uranyl acetate.

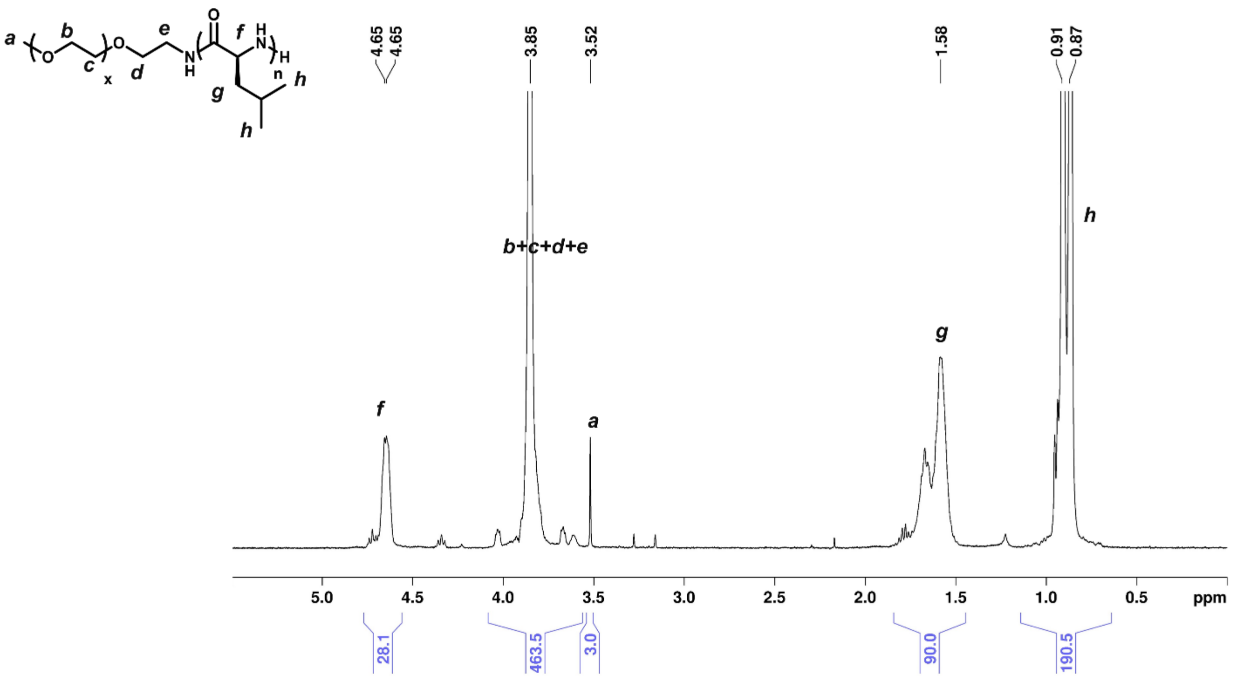

Figure S7. ^{1}H NMR spectrum of PEG_{114}-*b*-$PBLG_{32}$ in TFA-*d*.

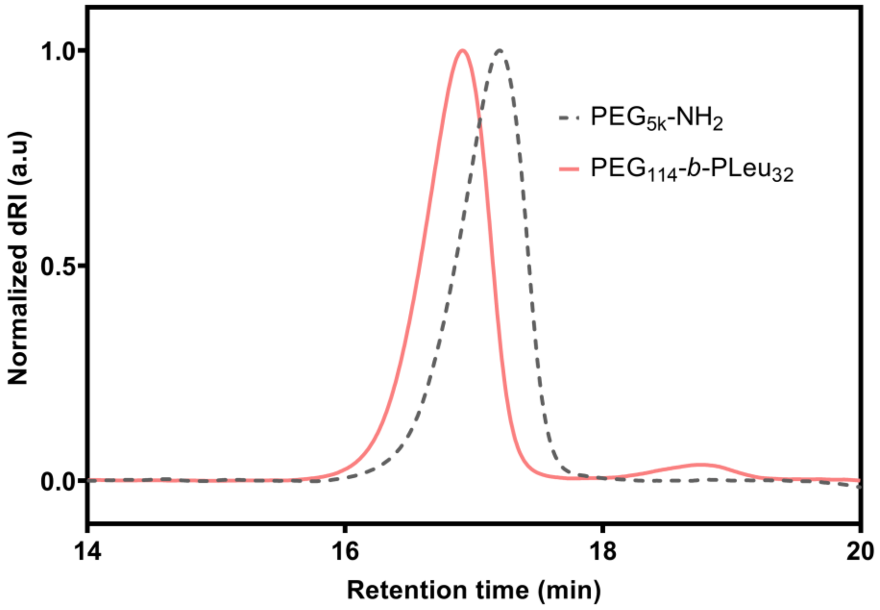

Figure S8. SEC traces of copolymer PEG_{114}-*b*-$PBLG_{32}$ compared to PEG_{5k}-NH_2 analyzed in HFIP + 0.05% KTFA (Differential refractive index detection, normalized).

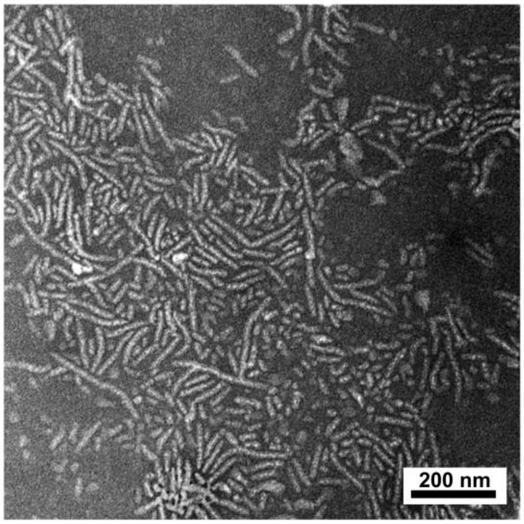

Figure S9. Representative TEM image of PEG_{114}-*b*-$PBLG_{32}$ nanoparticle suspensions, negatively stained with 1.2% uranyl acetate.

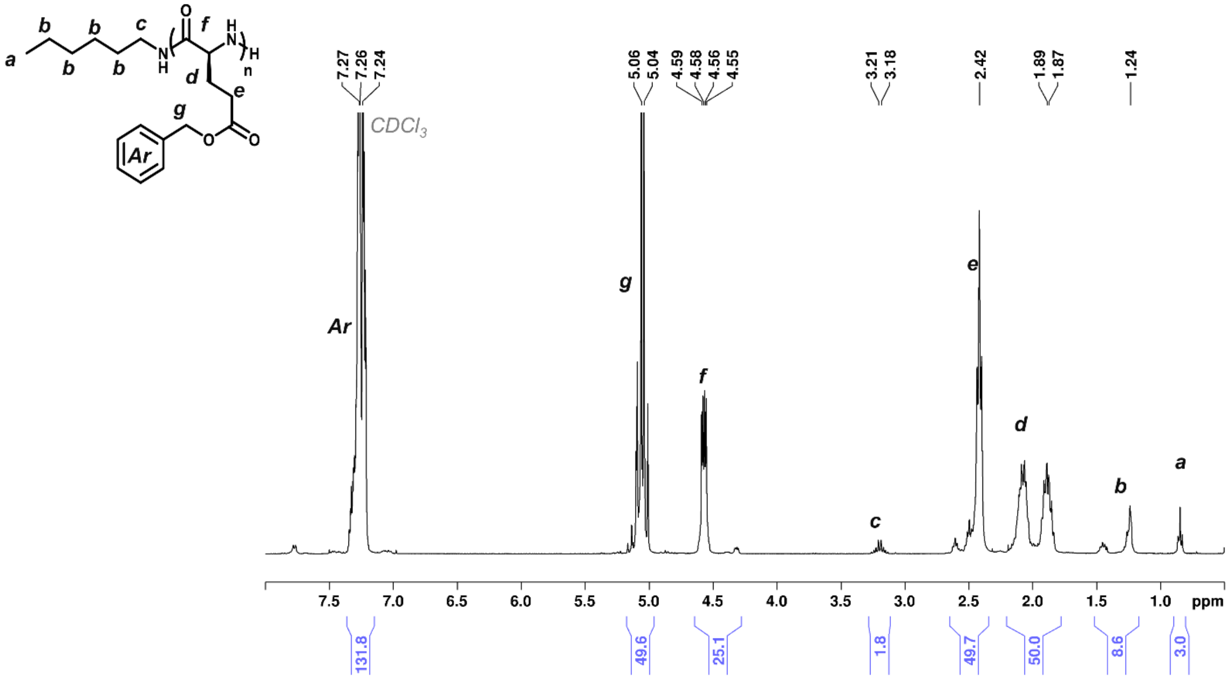

Figure S10. ^{1}H NMR spectrum of $PBLG_{25}$ in $CDCl_3$ + 15% TFA.

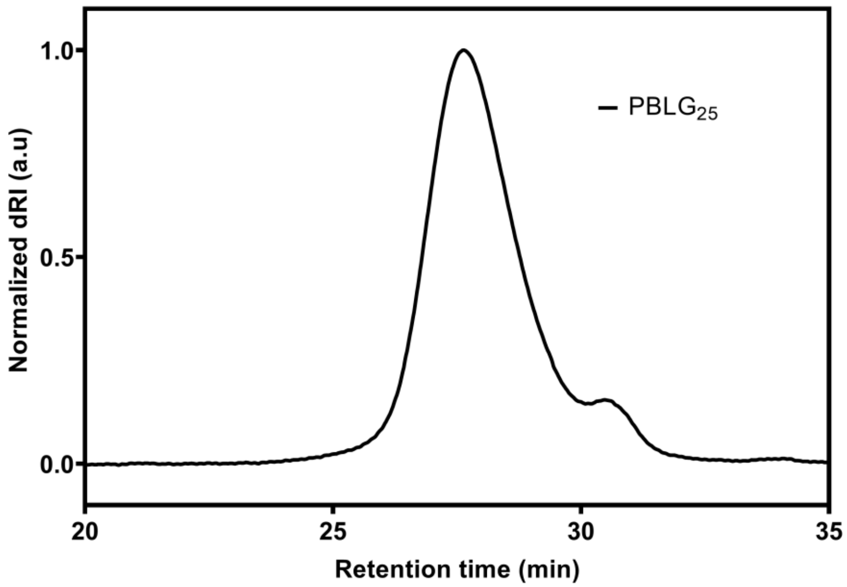

Figure S11. SEC traces of copolymer $PBLG_{25}$ analyzed in DMF + 1 $g.L^{-1}$ LiBr (Differential refractive index detection, normalized).

(B) Differential Scanning Calorimetry

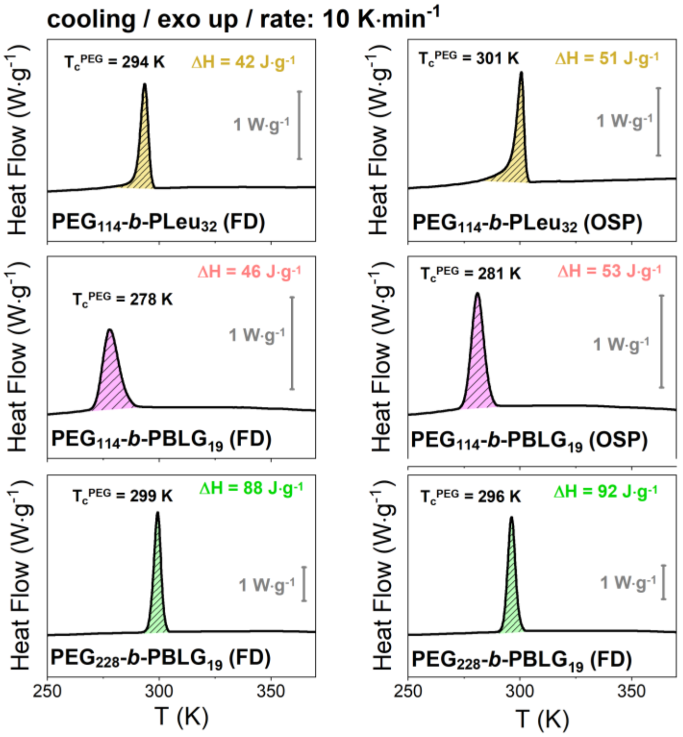

Figure S12. DSC traces of the copolymers, obtained during the cooling at a rate 10 K·min^{-1}. The shadowed areas represent the heat of fusion for the semicrystalline PEG for each sample. Crystallization temperatures are also indicated.

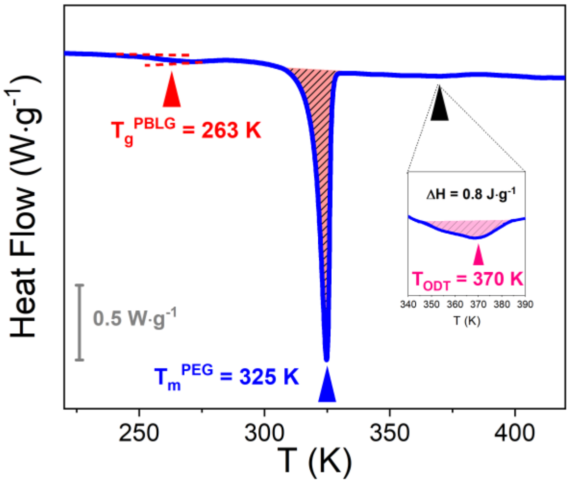

Figure S13. DSC trace of the PEG$_{114}$-*b*-PBLG$_{19}$ (OSP) copolymer, obtained during the heating at a rate 10 K·min^{-1}. The shadowed areas represent the heat of fusion for the semicrystalline PEG and the ODT transition. Starting from lower temperatures: blue arrow represents the glass temperature

of the PBLG block, purple arrow represents the melting temperature of the PEG block and pink arrow indicates the ODT transition temperature.

(C) Wide-Angle X-ray Scattering

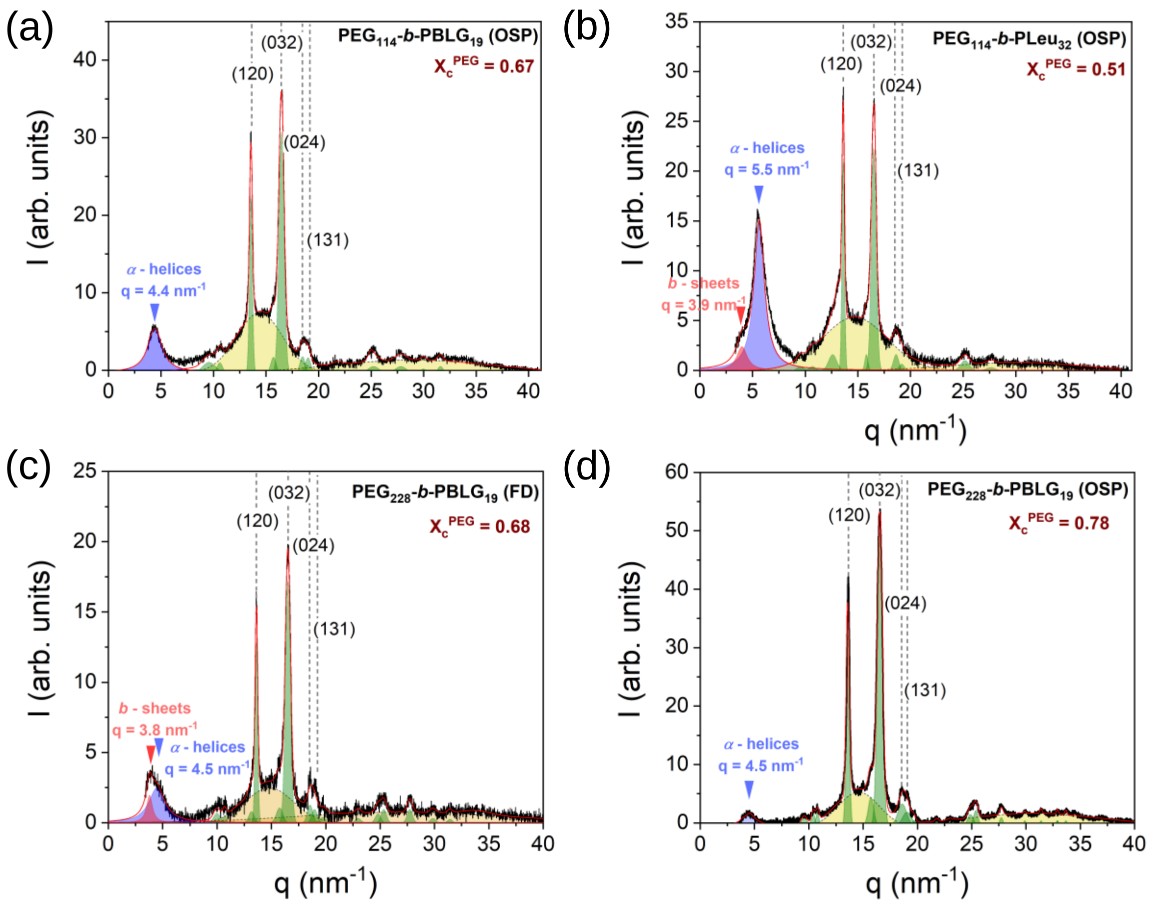

Figure S14. WAXS patterns of (a) PEG_{114}-*b*-$PBLG_{19}$ (OSP), (b) PEG_{114}-*b*-$PLeu_{32}$ (OSP), (c) PEG_{228}-*b*-$PBLG_{19}$ (FD) and (d) PEG_{228}-*b*-$PBLG_{19}$ (OSP).

(D) Dielectric Spectroscopy - Molecular Dynamics. Earlier combined studies by dielectric spectroscopy solid state NMR on PBLG as a function of molar mass revealed very rich dynamic behavior.[14] They have shown that the origin of the liquid-to-glass temperature observed in DSC was related to the low persistence length of the α-helical secondary structures. A broken network of hydrogen bonds responsible for the dynamic arrest at the glass temperature was also responsible for the low persistence of the helical segments. These processes were very distinct in dielectric spectroscopy. A fast segmental process with a strong temperature dependence (according to the

Vogel-Fulcher-Tammann -VFT- equation) was followed by a slower process associated with the relaxation of helical parts. The dielectric strength of the slower process was employed in calculating the persistence length of the helices.[13,14]

The corresponding DS results for the PEG_{114}-*b*-$PBLG_{19}$ (OSP) can be discussed with the help of **Figure S15**. For clarity, the processes below glass temperature of PEG have been omitted. Two segmental processes can be identified at T > T_g^{PEG}. The faster one corresponds to the segmental dynamics of the PEG block, while the slower one is ascribed to the segmental relaxation of the PBLG block. The two processes approach each other in the copolymer, revealing some degree of molecular mixing at the interface between the blocks. At temperatures above 293 K, the motion of the ions trapped in the crystalline PEG block is also evident for both PEG_{114}-*b*-$PBLG_{19}$ (OSP) and PEG_{114}-*b*-$PBLG_{19}$ (FD) copolymers. It can be seen that ions move faster in the latter case. This can be explained through two competing factors in the PEG_{114}-*b*-$PBLG_{19}$ (FD): the mixing of the PEG block with the slower PBLG block, which decreases ion mobility, versus the reduced crystallinity of PEG, that increases ion mobility. The experimental data suggest the predominance of the second factor. At higher temperatures, electrode polarization and Maxwell-Wagner-Sillars interfacial polarization mask any molecular processes (e.g. the one associated with the relaxation of PBLG α-helices). Nevertheless, the DS results revealed two segmental processes in the PEG_{114}-*b*-$PBLG_{19}$ copolymers and molecular mixing at the interface.

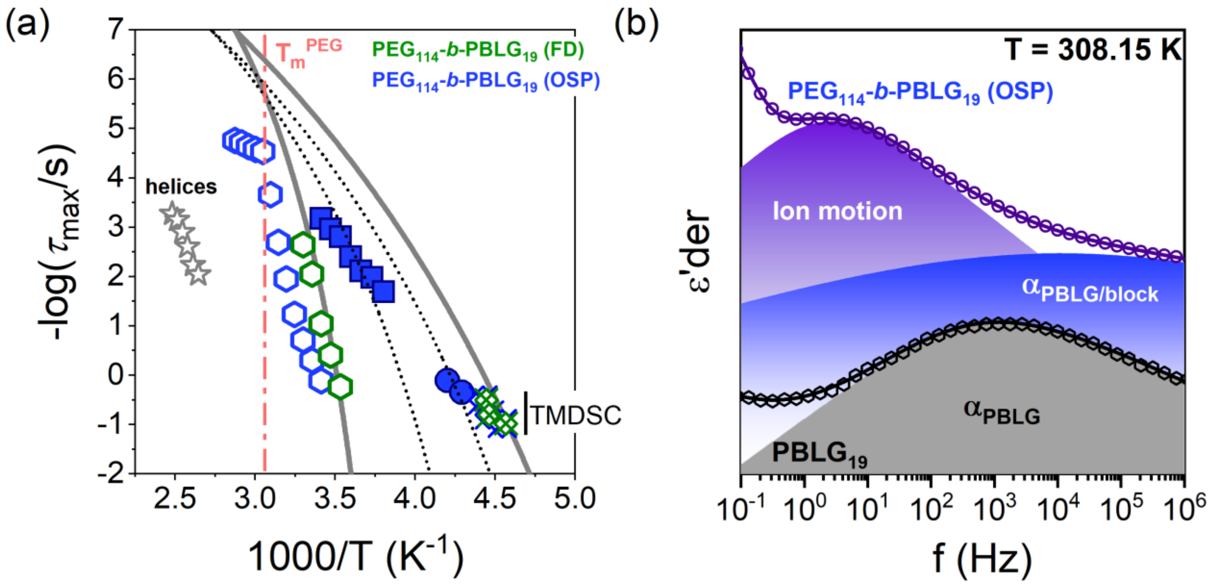

Figure S15. (a) Relaxation times as a function of the inverse temperature for the different processes of PEG_{114}-*b*-$PBLG_{19}$ (OSP) (blue) and PEG_{114}-*b*-$PBLG_{19}$ (FD) (green). Circles represent the segmental process of PEG, squares the segmental process of PBLG and hexagons the motion of ions. Doted black lines represent indicative fits to the PEG and PBLG segmental processes. Pink line indicates the melting temperature of the PEG block. Solid gray lines are simulation of the VFT function for the bulk PEG and PBLG homopolymers (Table S1), while gray stars represent the slow helix process in bulk PBLG. TM-DSC data are also presented with crosses. (b) Derivative of dielectric permittivity as a function of frequency for PEG_{114}-*b*-$PBLG_{19}$ (OSP) (circles), and bulk $PBLG_{19}$ (hexagons). Blue and purple areas of the copolymer correspond to simulations of the segmental process and the ion motion, respectively. The grey area represents the α process in the $PBLG_{19}$ homopolymer.

Table 1. Vogel – Fulcher – Tammann (VFT)* Parameters for the Segmental Relaxations obtained from DS for PEG_{114} and $PBLG_{19}$ homopolymers.

Sample	-log(τ_o/s)	B (K)	T_o (K)	T_g^{DS} (K)
PEG_{114}	-12	2570 ± 90	133 ± 4	212 ± 1
$PBLG_{19}$	-12	1265 ± 10	240 ± 1	278 ± 1

*VFT equation: $\tau_{max} = \tau_o e^{B/(T-T_o)}$
